# Supplementary material for: Embedded shape morphing for morphologically adaptive robots
Source: Nat Commun. 2023 Sep 27;14:6023. doi: 10.1038/s41467-023-41708-6 (PMC10533550; doi:10.1038/s41467-023-41708-6)
Supplement: Supplementary file 1 — Supplementary Information [file 41467_2023_41708_MOESM1_ESM.pdf]

Supplementary Information for

Embedded Shape Morphing for Morphologically Adaptive Robots

Jiefeng Sun[1,2,*], Elisha Lerner[1], Brandon Tighe[1], Clint Middlemist[1], and Jianguo Zhao[1,*]
[1]Department of Mechanical Engineering, Colorado State University, USA
[2]Department of Mechanical Engineering and Materials Science, Yale University, USA

[*]Authors for correspondence: jiefeng.sun@yale.edu and jianguo.zhao@colostate.edu.

This file includes:

Supplementary Notes

Supplementary Note 1. 2D bending shape-morphing module (SMM).

Supplementary Note 2. Analytical modeling of the sheathed TCA and shape-morphing modules.

Supplementary Note 3. Shape-morphing grippers.

Supplementary Note 4. Shape-morphing quadrupedal robot.

Supplementary Note 5. Shape-morphing amphibious robot.

Supplementary Note 6. Elementary shape-morphing modules.

Supplementary Figures

Supplementary Fig. 1. Comparison of our work with existing shape-morphing strategies.

Supplementary Fig. 2. Fabrication of the SMP spine.

Supplementary Fig. 3. Fabrication of the TCA.

Supplementary Fig. 4. Fabrication of the sheathed TCA and assemble the spine.

Supplementary Fig. 5. The minimal system.

Supplementary Fig. 6. Characterization of the SMP spine.

Supplementary Fig. 7. The schematic of the electric circuit for closed-loop control.

Supplementary Fig. 8. The schematics and results for the analytical model.

Supplementary Fig. 9. The design of the shape-morphing grippers.

Supplementary Fig. 10. Design of the shape-morphing quadrupedal robot.

Supplementary Fig. 11. Locomotion design of the quadrupedal robot.

Supplementary Fig. 12. The schematic of the electric circuit for the amphibious robot.

Supplementary Fig. 13. The gait of the amphibious robot.

Supplementary Fig. 14. The design of the twisting, 3D bending, and twisting and bending module.

Supplementary Fig. 15. The design of the discrete and grid surface module.

Supplementary Table

Supplementary Table 1. Comparison of shape-morphing schemes in existing studies.

Supplementary Notes

Supplementary Note 1. 2D bending shape-morphing module (SMM)

Supplementary Note 1.1. Fabricate the 2D shape memory polymer (SMP) spines

The fabrication process of the 2D shape memory polymer (SMP) spine is shown in Supplementary Fig. 2. We first 3D-printed the shape of the spine using an SLA printer (Prusa SL1S). Then we place the 3D-printed spine in a container and pour Mold Start 16 (Smooth-on Inc.) into the container to create a rubber mold. We then generate the SMP by mixing the Epon 828 and Diethylaminopropylamine (DMAPA) in a ratio of 100:6 by weight with a magnetic mixer under 45°C for 20 mins. The liquid SMP mixture is poured into the rubber mold that is preheated to 45 °C. The SMP is degassed in a vacuum oven under 45°C for 5 mins. A thermistor (EPCOS Inc., B57540G0503F000) is placed in the SMP liquid in the mold. After that, a flat rubber piece made of Sorta Clear (Smooth-on Inc.) is carefully placed on the top of the rubber mold to enclose the liquid SMP mixture. After curing under 75°C for 12 hours, the spines are demolded. A resistance wire (Nichrome, 36 Gauge) is then wrapped on the SMP spine at a distance of 2.5 mm. The embedded thermistor and the resistance wire are shown in Supplementary Fig. 2B.

Supplementary Note 1.2. Fabricate TCAs

The fabrication of TCAs follows the process described in Ref *(48)*. But we use a customized machine (an upgraded version compared with the machine in *(48)*) to fabricate TCAs with free strokes as shown in Supplementary Fig. 3. The main function of the machine is to fabricate a helical mandrel and coil a twisted fiber on the mandrel in the guiding groove. The machine has a wheel to fold the precursor thread, and twists the thread using two motors at the same time from its two ends. It can also be used to fabricate TCAs of different pitch angles.

For the specific configuration of TCA that we used in this work, 26 AWG copper wire is selected for the mandrel core, 32 AWG copper wire for the guide wire, and 235/36 dtex

4 ply conductive wire thread (V Technical Textiles, Inc. PN#200121235364HCB). The TCA machine is set to produce a helical angle of 22°. The manufacturing of a TCA can be split into five separate processes: building the mandrel, twisting the thread, coiling the twisted thread around the mandrel, annealing the TCA, and finally removing the guide wire from the TCA.

A more detailed tutorial video for fabricating the TCAs can also be found at https://youtu.be/tPyo5HDTpkw. The following paragraphs are detailed descriptions.

To make the mandrel, we start by cutting a 90 cm section of 26 AWG copper wire and twisting approximately 1 cm diameter loops on both ends. Connect the mandrel to motors 1 and 3. Twist a loop into the free end of the 32 AWG wire, attach it to the same side hook as the mandrel and unwind approximately 2.5 m from the spool. After running the guide wire through the lower pulley and back up towards the top of the machine, clamp the spool to the traveling plate that motor 2 is mounted to in a fashion so that it will not unspool more wire. Ensure 500 g mass is added to the plate that the pulley is mounted on to apply appropriate guide wire tension. With the speed selection set to medium, use the "mandrel only" function (just rotate the mandrel without moving the traveling guide) to secure the guide wire to the mandrel and position it to insert into the traveling wire guide. With the guide wire seated into the traveling guide and tensioner rubber bands on, increase the motor velocity to high and use the "coil" function to coil the guide wire around the mandrel. Make sure to closely monitor the machine and manually stop at the bottom of the TCA (before triggering the lower limit switch). Reduce the motor speed to medium and use the "mandrel only" function to secure the guide wire at the bottom of the mandrel, being careful not to relieve tension as that can cause an erroneous and nonuniform helical angle. After securing the guide wire, terminate it by cutting and tying off the excess.

Next, we will free motor 1 for the next operation by removing the mandrel from the motor and placing it on the hook beside motor 1. With the motor speed set to high, use the "home

travelers" function to bring motor 2 and the traveling guide back to their starting positions. The next process is to prepare and twist the thread. To make an 80 cm TCA, cut 3.6 m of conductive thread and tie small loops on both ends. Attach one end to motor 1 and after running the thread through the lower pulley, attach the other end to motor 2 and make sure the 500 g mass is on the lower pulley plate and that the thread is under tension. It is important to ensure that the machine is set to apply the appropriate amount of twist for the chosen length of natural thread as discussed in the previous section. After doing so, ensure the motor speed is set to high and use the "twist" function to begin twisting the thread. The machine will automatically stop when the appropriate amount of twist is inserted into the thread.

To coil the twisted thread around the mandrel, remove the mandrel from the hook and attach it to the motor on the opposite side as the thread loop. Use medium or low speed and the "mandrel only" function to secure the thread to the mandrel, place it into the traveling guide, and align it with the guide wire that is previously attached to the mandrel. On the high-speed setting, use the "coil" function to coil the twisted thread onto the entire length of TCA. Once completed, twist a loop in the mandrel at the top and bottom before removing it from the machine, so that the tread cannot untwist or uncoil from the mandrel.

Next is the annealing process, which is also dependent on the materials used and the configuration of the TCA being built. For our purposes, anneal in an oven for 2 hours at 185 °C. Finally, after the TCA has cooled off, we can remove the guide wire. This is done by untwisting the "locking loops" from the mandrel and replacing it back onto motors 1 and 3. Next, cut the guide wire and hold the end with pliers or by hand while using the "mandrel only" function to unwind the guide wire. The TCA is now ready for use or storage. Typically we leave the TCA on the mandrel core while storing it to prevent deformation due to viscoelastic creep.

Supplementary Note 1.3. Fabricate sheathed TCAs

Supplementary Fig. 4A shows the fabrication of the elastic tube for sheathed TCAs. We place carbon fiber rods (diameter 1.2 mm) into the channels of a 3D printed mold and pour liquid Ecoflex-50 (Smooth-On Inc.) into the mold. After Ecoflex-50 is cured, we cut the extra part and leave the tube. As shown in Supplementary Fig. 4B, we first cut a TCA and connect the two ends of the TCA with two copper wires as electrical leads. Then the TCA is inserted into the tube, and the two ends are glued to the tube with the silicone glue (Sil-Poxy, Smooth-on Inc.). The cross section of the sheathed TCA is shown in Supplementary Fig. 4C.

Supplementary Note 1.4. Assemble sheathed TCAs and the Spines for the 2D bending SMM

Since the sheathed TCA is soft, we design a specialized jig to restrain their shapes while assembling it to the spines. Supplementary Fig. 4D shows the setup to assemble the 2D bending SMM. The gig has a groove of a U-shape. We first arrange the sheathed TCA into the groove of the jig. Then the spine is placed on the sheathed TCA in the jig after silicone glue is applied to the protrusion. During the curing of the glue, some weights are placed on top of the assembly to apply pressure, which ensures a better connection between the spine and the sheath.

Supplementary Note 1.5. A Minimal system for the 2D Bending SMM

Our shape-morphing scheme can be realized using small and common off-the-shelf electronics in a self-contained manner because no bulky components such as pumps or magnetic coils are required. To demonstrate this, we build a minimal system using an 11.1 V battery, a microcontroller, and a motor driver to control voltages applied to both the TCA and the resistance wire wrapped around the SMP spine in Supplementary Fig. 5A. The size of the minimal system's control unit is 7 × 8 cm and weighs 25 g without a battery. Additional sensors (e.g., a current sensor to measure the TCA's electrical resistance) can be added to facilitate the control process, but all of them can be achieved in a self-contained manner. Such self-containment is particu-

larly suitable for robot locomotion in outdoor environments when access to external equipment is difficult or impossible. As shown in Supplementary Fig. 5B, the minimal system consists of a control and power unit and a 2D bending SMM. The control and power unit consists of a dual-channel motor driver (DRV8833, Texas Instruments), an 11.1 v 300 mAh battery (22 g), and a microcontroller (Arduino Pro Mini).

Supplementary Note 1.6. Characterization

The dynamic mechanical analysis for the SMP spine is conducted using SEIKO SII EXSTAR 6100 DMS. The storage modulus, the loss modulus, and the loss factor are shown in Supplementary Fig 6A. The glass transition temperature of the polymer is around 100 °C.

The comparison of the natural cooling and forced cooling using a fan is shown in Supplementary Fig. 6B. We heat the spine to about 110 °C under no-wind conditions at room temperature. The natural cooling process and the forced cooling process are both at room temperature. A fan is used for the force cooling process with a measured wind speed (0.8 m/s). It takes respectively around 14 s and 32 s for the spine to use natural cooling and forced cooling to recover over 95% of its stiffness (80 °C). The forced cooling is more than two times faster than natural cooling.

To measure the relationship between the bending angle and the resistance (Fig. 3e in the manuscript), a constant voltage of 10 V is applied to the module when the spine is softened, and the shape of the module is recorded as a video. The bending angle of the module is extracted by three marker points at the two ends and the middle using Tracker software (https://physlets.org/tracker/). The fitted polynomial shown in Fig. 3e is

$$\theta = 4.88R^3 - 277.86R^2 + 5288.27R - 33656.72,$$

where θ is the bending angle and R is the resistance of the TCA.

To demonstrate the module can be actuated very fast, the shape-morphing module is quickly

actuated using the voltage of 12.5, 18.75, and 25 V, after the spine is softened (Supplementary Fig. 6C). For 25 V, the module can bend around 250° in 1 sec.

Supplementary Note 1.7. Open-loop control

We achieve the open-loop control by applying a constant voltage to the TCA so that the module will reach a desired steady-state angle. After reaching the angle, it is crucial to maintain that angle for a specific period to allow the SMP spine to cool down and stiffen. Stopping the actuation of the TCA too early before the spine completely stiffens will allow the spine to bend back, resulting in a smaller holding angle. On the other hand, continuing to actuate the TCA when the spine becomes rigid may permanently damage the TCA. Therefore, it is essential to properly control the sheathed TCA to steadily hold the desired angle when the SMP spine stiffens.

To control the bending angle of the SMM, we first identify when to stop heating the spine and actuating the TCA. Such an approach is based on the characterization of the module by applying different constant voltages Ut (0.5 to 4 V with a step size of 0.5 V) to the TCA to obtain the module's steady state bending angles when the SMP spine is soft (Fig. 3c). We consider the module to have reached a steady state when the bending angle changes less than a small angle (we use 3° here) for a sufficiently long time (we use 32 s here). With these characterization results, we can employ open-loop control to morph the module to a desired angle by applying a constant Ut to the TCA corresponding to a specific steady-state angle. For instance, if we want to morph the module to 140° (corresponds to U_t = 3 V from Fig. 3c), we can first soften the SMP spine, then apply a constant Ut = 3 V to the TCA. Based on the curve in Fig. 3c, we can start to stiffen the SMP spine at 138 s but keep applying 3 V to the TCA. Finally, we stop actuating TCA after another 32 s.

Supplementary Note 1.8. Closed-loop control

We implement the closed-loop control using a customized circuit (the schematic shown in Supplementary Fig. 7) to control the bending angle for the 2D bending SMM. In the circuit, we use an Arduino Uno as a controller, a 12-bit DAC module (MCP4725) to generate $0 - 5$ V voltage that can be amplified using an LM2596 buck converter to control a TCA, and a motor driver (MC33926) is used to drive the resistance wire wrapped on the spine. A voltage-current sensor (INA219) is used to measure the TCA's electrical resistance. Note that this circuit differs from our previous work *(47)* since it can always generate a continuous DC voltage instead of a PWM voltage. The continuous DC voltage will greatly reduce the INA219's measurement error compared with a PWM voltage input, especially when the duty cycle of the PWM signal is low.

We implemented the following PI controller for the closed-loop control of the bending angle of the shape-morphing module.

$$V = K_p(R - R_d) + K_i \int (R - R_d)$$

where V is the voltage applied to the TCA, R_d is the desired resistance corresponding to the desired bending angle. For this controller, we choose the values $K_p = 150$ and $K_i = 1$ to generate good control performance (e.g., small overshoot, short rising time). A digital filter based on a simple moving-average algorithm (window counts 10) is implemented to reduce high-frequency uncertainties. The TCA's resistance is directly calculated by the measured voltage and current at the same time instead of the voltage we applied to the TCA.

Estimation of morphing speed: Different from a continuous moving system, a shape-morphing system will start to stiffen whenever a very small steady-state error is accomplished (e.g., $\pm 3°$). Because a large steady-state error may either damage the actuator or the variable-stiffness spine when the spine starts to stiffen. Therefore, we use settling time t_s to compare the two control methods, which is the time when the angle enters a small bound ($\pm 3°$) and stiffening of the spine can start. Figure 3f of the manuscript shows that $t_s = 10$s and $t_s = 138$

s respectively for open and closed loop control. Therefore, our closed-loop control is 10 times faster than the open-loop control method.

Supplementary Note 1.9. Comparisons between TCAs and other actuators for shape-morphing purposes

In this note, we compare TCAs with other actuators that can be driven by electricity and do not require larger peripherals, i.e., dielectric elastomer actuators (DEAs) and hydrogel actuators.

TCAs VS. DEAs

Both actuators are driven by electricity, but a key difference lies in the required voltage levels: dielectric ones are generally driven by a high voltage (hundreds to kilo Volts), whereas TCAs only require a low voltage (5-20 V). This means that dielectric systems require an additional high-voltage converter. While low-power (< 1 W) converters can be compact, as the power requirement increases, the size and weight of these converters escalate nonlinearly. For example, the Q series of the widely used EMCO Series DC – HV DC Converter, which operates at a power of 0.5 W, weighs a mere 4.25 g. However, an F series model operating at a power of 10 W weighs 142 g, approximately the weight of an iPhone 13. Therefore, in terms of bulky peripherals, a TCA-driven system generally holds an advantage over dielectric ones, since it does not require an additional high-voltage converter. This advantage becomes more pronounced for larger-scale systems.

Further, our scheme has another advantage over DEA ones in terms of “versatile embeddedness”. The “embeddedness” in this work does not only mean the system could be driven with small peripherals but also mean how “versatile” the design could be – how difficult to realize many different shapes/motions. To realize “versatile” motion, DEAs have to be fabricated into configurations, e.g., stacked, rolled, tubular, and bending etc. There are methods to program the Gaussian curvature of a DEA surface, but the fabrication process is complicated. A TCA could

be used as a basic element arranged in different shapes to achieve programmable shapes due to the linear contraction and flexibility. For example, twisting motion and 3-way bending motion can be realized by arranging TCAs in helical and three parallel lines.

Finally, there is no shape-morphing scheme with DEAs that can achieve shape actuation, sensing, and locking at the same time. A detailed summary of the DEA-driven morphing system is presented in Supplementary Table 1.

TCAs VS. Hydrogels

TCAs have a much larger energy work density than current typical hydrogels. The energy density of current typical (osmotic-driven) hydrogels is 10^{-2} kJ/m^3. Even with energy storage, it can only reach an energy density of 15.3 kJ/m^3, while a nylon-based TCAs' energy density is 2000 kJ/m^3, which is more than 100 times the strongest gels. The higher energy density of the artificial muscle can facilitate the "embeddedness" of the artificial muscle, and will especially benefit a shape-morphing system because the system does not need to carry heavy actuators while the actuation only works infrequently.

TCAs could be easily driven by electricity and can be individually actuated. Our current TCAs are made of commercially available nylon threads, which are silver coated, making them electrically conductive. Further, we can embed multiple TCAs into a body to individually actuate them. In contrast, to actuate gels, a uniform external stimulus is generally required (e.g., temperature, electric, magnetic, etc.)

Supplementary Note 2. Analytical Modeling of the sheathed TCA and 2D/3D bending SMMs

An analytical model will allow us to predict the morphed shape of a shape-morphing module with a given electricity input (i.e., the voltage applied to the TCA) to provide insights into the design of the SMM. Here we use the 2D SMM as an example.

The general modeling procedure is as follows. We first predict the sheathed TCA's tem-

perature for a given voltage input using a thermal model. The temperature can be input into a thermal-mechanical model of TCAs using Castigliano's Second Theorem (CST) to predict TCA's actuation. The 2D bending SMM, once softened, can be modeled using the Cosserat rod theory. Finally, we solve the deformation by simultaneously solving the TCA actuation and the spine deformation *(49)*.

Supplementary Note 2.1. Modeling of a sheathed TCA

The temperature of a sheathed TCA with respect to time can be calculated using a thermal model by simplifying the problem into 2D (a cross section) and considering the length of a sheathed TCA is much larger than its diameter (Supplementary Fig. 8A). The TCA and the sheath are considered as two solid rings. The TCA transfers heat to the sheath through conduction, and the sheath dissipates energy through air convection. A toolbox (ThermalModel) in Matlab is used to solve the heat transfer problem. We only need the steady-state temperature to predict the final shape.

The contraction displacement of a TCA can be considered as coming from two parts: the change of temperature and external force F_e *(49)*.

$$\delta_a = A_{cst}\Delta\bar{\theta}^{\mathsf{h}} + \frac{1}{K_{cst}}F_e \tag{1}$$

where the first part captures the temperature's effect with $\Delta\bar{\theta}^{\mathsf{h}}$ is the unit untwisting of a twisted fiber used to fabricate TCAs with respect to temperature. A polynomial can be used to approximate $\Delta\bar{\theta}^{\mathsf{h}} = -0.0161T^2 + 0.3338T - 7.7311$ based on our previous work *(49)*, and

$$\frac{1}{K_{cst}} = l_t\left(\frac{r^2\cos^2\alpha}{G_t J_t}\right) \tag{2}$$

$$A_{cst} = l_t r\cos\alpha \tag{3}$$

where r is the TCA's diameter, l_t is the twisted fiber's length, A_t is the cross section area of the twisted fiber, and α is the pitch angle of the TCA. Note that based on the small deformation

assumption, all the variables are close to their values of the reference states, for example, $\alpha \approx \alpha^*$. We also ignore the bending, shear, and extension strain of the TCA, and use constant G_t for simplicity. All the parameters can be found in our previous work *(49)*.

For sheathed TCAs, the elastic sheath will resist the contraction by increasing the actuator's stiffness. We can replace K_{cst} with K_{sha} where $K_{sha} = K_{sh} + K_{cst}$. $K_{sh} = 0.23$ N/mm is the stiffness of the elastic sheath, which is calculated based on the sheath's geometry and Young's modulus (E = 0.1 MPa). In this case, the contraction for sheathed TCA can be obtained as

$$\delta_{sha} = \frac{A_{cst}K_{cst}}{K_{sha}}\Delta\bar{\theta}^{\mathsf{h}} + \frac{1}{K_{sha}}F_e \tag{4}$$

Note that a TCA's force includes both the force from the spine and the sheath:

$$F_{tca} = F_e + K_{sh}\delta_{sha} \tag{5}$$

Supplementary Note 2.2. Kinetostatics for SMMs

We model the SMP spine that is softened and actuated by TCAs using Cosserat Rod theory to provide a general modeling framework. In Cosserat theory, bodies are modeled as a collection of infinitesimal rigid bodies (IRBs) rather than point particles, and thus each IRB has both position and orientation rather than just position. As shown in Supplementary Fig. 8B, for a given rod, we define the centerline, $s \in [0, L]$, as the curve passing through the centroids of all the cross sections where L is the length of the rod in its reference configuration. At every point s along the centerline, we establish a body frame for the cross section. The z axis of the frame is tangent to s, and the x and y axes are assumed to be aligned with the principal axes of the cross sections. Each body frame has a rotation and a translation relative to the global (fixed) frame. We describe the position and orientation of all the IRBs along the centerline using a rotation matrix $R(s) \in SO(3)$ and a position vector $p(s) \in \mathbb{R}^3$.

We write the system of ODEs using R, $\boldsymbol{p}$, and $\boldsymbol{\xi}$ as state variables *(49)*.

$$R' = R\widehat{\boldsymbol{u}} \tag{6}$$

$$\boldsymbol{p}' = R\boldsymbol{v} \tag{7}$$

$$\boldsymbol{\xi}' = K^{-1}(ad_{\boldsymbol{\xi}}^T K\Delta\xi - \bar{\boldsymbol{W}}_e) + \boldsymbol{\xi}'^* \tag{8}$$

where $'$ is the derivative with respect to s, and $\boldsymbol{\xi} = [\boldsymbol{u}^T, \boldsymbol{v}^T]^T \in \mathbb{R}^6$ is the spatial twist (strain) representing the relative configuration change between adjacent cross sections along the centerline, with $\boldsymbol{u}, \boldsymbol{v} \in \mathbb{R}^3$ the angular and linear strain component, respectively. The 'hat' operator $\widehat{\cdot}$ is a mapping from $\mathbb{R}^3$ to $\mathfrak{so}(3)$ or $\mathbb{R}^6$ to $\mathfrak{se}(3)$, e.g., $\widehat{\boldsymbol{\xi}} = \begin{bmatrix} \widehat{\boldsymbol{u}} & \boldsymbol{v} \\ \boldsymbol{0} & 0 \end{bmatrix}$, $\bar{\boldsymbol{W}}_e = \begin{bmatrix} \bar{\boldsymbol{l}}^T, & \bar{\boldsymbol{f}}^T \end{bmatrix}^T$ is the distributed external wrench with $\bar{\boldsymbol{l}}, \bar{\boldsymbol{f}} \in \mathbb{R}^3$ as the moment, force per unit arclength applied to the centerline in the body frame, $ad_{\boldsymbol{\xi}} = \begin{bmatrix} \widehat{\boldsymbol{u}} & 0 \\ \widehat{\boldsymbol{v}} & \widehat{\boldsymbol{u}} \end{bmatrix}$ is the adjoint representation of the spatial twist $\boldsymbol{\xi}$. $\boldsymbol{W}_i = \begin{bmatrix} \boldsymbol{m}^T, & \boldsymbol{n}^T \end{bmatrix}^T$ is the internal wrench in the body frame with $\boldsymbol{m}, \boldsymbol{n} \in \mathbb{R}^3$ as the internal moment and force in the body frame. $K = \textbf{diag}[EI_x, EI_y, GJ, GA_t, GA_t, EA_t]$ is the diagonal stiffness matrix for bending, torsion, shear, and extension. E and G are Young's modulus and shear modulus, respectively. A_t is the cross section area of the rod, I_x, I_y are the second moment of area with respect to x and y axis, and $J = I_x + I_y$ is the polar moment of inertia of the rod's cross section about its centroid.

External distributed wrench comes from the artificial muscle and gravity

$$\bar{\boldsymbol{W}}_e = \bar{\boldsymbol{W}}_{grav} + \bar{\boldsymbol{W}}_a \tag{9}$$

where

$$\bar{\boldsymbol{W}}_{grav} = \rho A \begin{bmatrix} \boldsymbol{0}_{3\times1} \\ R^T \boldsymbol{g}_r \end{bmatrix}, \tag{10}$$

is the distributed gravitational force in the global frame and $\boldsymbol{g}_r = [0, 0, -9.81]^T$ is the gravitational vector. $\bar{\boldsymbol{W}}_a$ is the distributed wrench due to the artificial muscle TCA's tension force.

The initial and distal boundary conditions need to be adjusted as

$$\boldsymbol{W}_i(0) = \boldsymbol{W}_{i,0} - \boldsymbol{W}_a \tag{11}$$

$$\boldsymbol{W}_i(L) = \boldsymbol{W}_a + \boldsymbol{W}_{ext} \tag{12}$$

where $\boldsymbol{W}_{i,0}$ is the initial internal wrench if the TCA is connected to the ground, $\boldsymbol{W}_a$ is the sum of point wrench exerted by TCAs, and $\boldsymbol{W}_{ext}$ is the sum of explicit external wrench applied to the distal end.

Supplementary Note 2.3. Sheathed TCA force mapped to the spine

A sheathed TCA is glued onto the protrusions of the SMP spine. The TCA can move in the sheath. The ends of the TCA are always fixed to the spine, typically at the ends. In this case, the ith TCA will exert a point wrench due to the tension. This can be written in the body frame as:

$$\boldsymbol{W}_{a,i} = Ad_g^T \boldsymbol{W}_{a,i}^g = -F_a \begin{bmatrix} \widehat{r}_a R^T \boldsymbol{t}_a \\ R^T \boldsymbol{t}_a \end{bmatrix} \tag{13}$$

where $F_a = 2F_e$ since we use two TCAs (U-shape) for a single module. Due to the short distance between protrusions, the force applied by the TCAs can be considered the same as a uniformly distributed force. If there are n TCAs, the total distributed wrench will be

$$\bar{\boldsymbol{W}}_a = \bar{A}\boldsymbol{\xi}' + \bar{B} \tag{14}$$

$$\boldsymbol{W}_a = \Sigma_{i=1}^n \boldsymbol{W}_{a,i} \tag{15}$$

where $\bar{A} = \Sigma_{i=1}^n \bar{A}_i$ and $\bar{B} = \Sigma_{i=1}^n \bar{B}_i$, and

$$\bar{A}_i = -F_a \begin{bmatrix} -\widehat{\boldsymbol{r}}_a P \widehat{\boldsymbol{r}}_a & \widehat{\boldsymbol{r}}_a P \\ -P\widehat{\boldsymbol{r}}_a & P \end{bmatrix}, \bar{B}_i = -F_a \begin{bmatrix} \widehat{\boldsymbol{r}}_a \bar{C}_i \\ \bar{C}_i \end{bmatrix}$$

$$\bar{C}_i = b - P(\widehat{\boldsymbol{r}}'_a \boldsymbol{u} - \boldsymbol{r}''_a)$$

where $b = P[\widehat{\boldsymbol{u}}(\boldsymbol{v} - \widehat{\boldsymbol{r}}_a \boldsymbol{u} + \boldsymbol{r}'_a)]$, $P = -R^T \frac{(\widehat{\boldsymbol{p}'}_a)^2}{\|\boldsymbol{p}'_a\|^3} R$.

Both $\bar{A}_i \in \mathbb{R}^{6\times6}$ and $\bar{B}_i \in \mathbb{R}^6$ are independent of $\boldsymbol{\xi}'$, but dependent on $\boldsymbol{\xi}$. It is simple to use $\boldsymbol{\xi}$ instead of $\boldsymbol{W}_i$ as the state variable because of the dependence of $\bar{\boldsymbol{W}}_a$ on $\boldsymbol{\xi}$ and $\boldsymbol{\xi}'$.

Supplementary Note 2.4. Sheathed TCA coupling to the Spine

When glued to the spine, both a TCA's displacement and tension force are coupled to the body's deformation. The displacement of a TCA can be calculated from the positions of the TCA coupled to the spine.

$$\delta_{sha} = \int_0^L \|\boldsymbol{p}_a'\| ds - \int_0^L \|(p_a^*)'\| ds \tag{16}$$

where δ_a is the displacement of the sheathed TCA, and this is the difference of the TCA's arc length between the current configuration and the original configuration.

Combining (16) and (5), we can have a compatibility equation for the TCA

$$\int_0^L \|\boldsymbol{p}_a'\| ds - l^* - (\frac{A_{cst}K_{cst}}{K_{sha}}\Delta\bar{\theta}^{\mathsf{h}} + \frac{1}{K_{sha}}F_e) = 0. \tag{17}$$

Plugging (14) in (9) and (8), and rearrange to make $\boldsymbol{\xi}'$ explicit, we have

$$\boldsymbol{\xi}' = (K + \bar{A})^{-1}(ad_{\boldsymbol{\xi}}^T K\Delta\xi - \bar{B} - \bar{\boldsymbol{W}}_{grav} + K\boldsymbol{\xi}'^*). \tag{18}$$

Rearranging (11) and (12), we can obtain

$$\boldsymbol{\xi}(0) = \boldsymbol{\xi}_0 - K^{-1}\boldsymbol{W}_a \tag{19}$$

$$\boldsymbol{\xi}(L) = K^{-1}(\boldsymbol{W}_a + \boldsymbol{W}_{ext}) + \boldsymbol{\xi}^* \tag{20}$$

where $\boldsymbol{\xi}_0$ is the initial strain if the module's one end is fixed.

A single module driven by a sheathed TCA can be fully defined by the system of ODEs (6), (7) and (18) with compatibility equation (17) and boundary condition (19) and (20).

Supplementary Note 2.5. Numerical implementations

Sheathed TCA actuation force F_a is an implicit variable that is required both for the boundary condition and for the ODEs. A shooting method can be used to solve the boundary value

problem with the unknown/implicit force. The method starts by guessing the initial strain $\boldsymbol{\xi}_0$ and the actuation forces F_a using the trust-region-dogleg method. Then the ODEs are integrated from $s = 0$ to $s = L$ using a standard library code (e.g., $ode45$ in Matlab), during which the distributed wrench and point wrenches will need to be calculated. Finally, the boundary conditions and the compatibility equation are checked. If a residual error ϵ as in (21) is within a specified tolerance, the algorithm stops and returns the results; otherwise, new guess values will be generated, and the process repeats until the boundary conditions and the compatibility equations are satisfied.

$$\boldsymbol{\epsilon} = \begin{bmatrix} \int_0^L \|\boldsymbol{p}_a'\| ds - l^* - (\frac{A_{cst}K_{cst}}{K_{sha}} \Delta\bar{\theta}^{\mathsf{h}} + \frac{1}{K_{sha}} F_e) \\ K^{-1}(\boldsymbol{W}_a + \boldsymbol{W}_{ext}) + \boldsymbol{\xi}^* \end{bmatrix} \tag{21}$$

With the simulation method, we simulated the case with constant voltages for 3 mins and use the final angle as the steady-state angle to plot Fig. 3d. We observed discrepancies for voltage 2, 2.5, and 3 V. The reason for the discrepancy is likely the inaccurate modeling of the sheathed TCA's mass distribution. The gravity from the mass influences differently before and after (increased bending) the module bends 90°. It decreases the bending angle before 90° and increases the angle after 90°. This is the reason why the discrepancies happen after the module bends over 90° In the simulation, we can only apply gravity to the centerline of the rod (the spine). Although we increase the density of the spine to incorporate the weight of the sheathed TCAs and the protrusions, the added mass is not at the right location but concentrated on the spine.

Supplementary Note 2.6. Influence of the protrusion height

The analytical model can be used to offer insights into the design of SMMs with different parameters. For example, the influence of the protrusion height can be determined using the model. As shown in Supplementary Fig. 8C, we predict the steady-state bending angle by varying the protrusion height (from 3 mm to 9 mm) for different constant voltages applied to

the TCA. The results show that if the distance is larger, the steady-state bending angle for a specific voltage will be smaller. However, if the distance is too small, the module can have a larger steady-state bending angle, but the required force may exceed the maximum capability of the TCA (0.6 N). Based on the results, we want to obtain enough bending angle ($>180°$) and be as safe as possible. Therefore, we choose a distance of 6 mm, leading to the actual protrusion height of 5 mm with the sheath (radius $\sim$1 mm).

Supplementary Note 3. Morphing grippers

Supplementary Note 3.1. Fabrication of the morphing grippers

The base of the two types of morphing grippers is a modified 2D bending SMM. To attach a holder to the center of the module, when we wrap the resistance wire, we skip the middle part (about 10 mm) to glue the holder to the spine.

For the morphing gripper 1, the compliant spines of the compliant fingers are directly 3D printed with Nylon filaments, and the sheathed TCAs are glued onto the protrusion, which has a similar design as the SMM. A 3D printed finger connector connects the compliant finger with the shape-morphing module using press fit as shown in Supplementary Fig. 9A.

The morphing gripper 2 is fabricated by arranging two 2D bending SMMs in a cross shape. Again, the middle part is skipped when the resistance wire is wrapped on the two modules. The four rigid fingers are directly 3D printed with Nylon filaments and assembled onto the two SMMs as shown in Supplementary Fig. 9B.

Supplementary Note 3.2. Control of the morphing grippers

For the morphing gripper 1, the shape-morphing base is characterized and controlled using closed-loop control as detailed in Supplementary Note 1.9. The required bending angle of the base is estimated based on the size of the objects to make sure that the compliant fingers can touch the object after morphing. For example, to pinch a ping-pong ball of 40 mm, the base is

morphed to 180°. Once the morphing is finished, the fingers are actuated to apply pressure on the objects to pick them up. The way to control the fingers is documented in *(47)*.

For the morphing gripper 2, the two pairs of fingers are characterized to make sure that they can be precisely controlled. The required bending angles are estimated, and the two pairs of fingers are controlled to morph into the shape of the objects. For example, to cage the mouse, one pair of fingers is bent to 93°, and the other pair is bent to around 122°.

Supplementary Note 3.3. Power estimation of the grippers

In Fig. 4e, we compare the power consumption of morphing gripper 2 with a normal soft gripper. Here we describe how our estimation is carried out for caging the mouse as an example.

Energy for shape morphing: The two pairs of fingers bend respectively 93° and 122° for caging the mouse. For a single module, this energy includes two parts: heating up the SMP spine and actuating the TCA while the spine is stiffening. We calculate the energy consumption by dividing the morphing process into two stages as shown in Supplementary Fig. 9. In the first stage, the energy required to soften the spine can be estimated as

$$E_s = \frac{U_s^2}{R_s} t_s \tag{22}$$

where $R_s = 55$ ohms, $U_s = 25$ V, and $t_s = 20$ s are, respectively, the resistance, the voltage applied to the resistance wire, and the time during which the power is applied to soften the spine.

In the second stage, the TCA is actuated to bend to the desired angle in 2-3 s and the angle was maintained with the closed-loop control. We recorded the voltage and resistance to calculate real-time power. The consumed energy for actuating the TCA in ith finger $E_{ai}(t)$ can be calculated by integrating the power with respect to time. In Fig. 4e of the manuscript, we plot the energy consumption for two pairs of fingers when the second stage starts. The starting point for the gripper (two fingers) is $2E_s$ (a constant), and during $t = 0 - 32$ s, it is

$2E_s + E_{a1}(t) + E_{a2}(t)$, where E_{a1} and E_{a2} are functions of time t. After 32 s, the pairs of two fingers hold the configuration.

The soft gripper: A soft finger does not need to soften a spine. It starts at 0, and during $t = 0 - 32$, the energy consumed is $E_{a1}(t) + E_{a2}(t)$. After 32 s, it requires continuous energy input. We assume that the input power is the same as the power at the end of 32 s for simplicity.

Supplementary Note 4. Shape-morphing quadrupedal robot

Supplementary Note 4.1. Mechanical design

The robot has two main components as in Supplementary Fig. 10A. 1) the morphing body: three 2D bending SMMs in parallel, 2) four legs. Two 3D-printed body connectors are used to connect the SMMs and the four legs, initially, in a flat shape. The four legs and the three SMMs are designed to be press-fit together on the body piece to avoid the weight and complexity that come with using hardware.

The body connectors were designed so that the robot would be symmetric around its sagittal and coronal planes. The two identical body connectors are 95 mm in length and work together to hold three SMMs in parallel. There are channels to connect the body mount clips aligned to hold the legs 75° from the body piece (15° from parallel with the coronal plane), which increases the size of the robot base and acts to stabilize it from tipping.

As shown in Supplementary Fig. 10B, each leg is a continuum bending manipulator that can bend into any direction driven by three TCAs of made length 105 mm. The spine of the leg is made of a carbon fiber rod with a diameter of 0.3 mm to ensure that the leg has only a bending motion. Seven plates are evenly distributed along and fixed to the spine, and TCAs run through the holes in the plates. The three TCAs are connected together to a common ground wire that also runs through a hole in the plates. A plastic shoe is used to cover the end of the

leg to protect the electrical connection of the TCAs.

Supplementary Note 4.2. Electrical system

An electrical system is designed to be used manually by an operator. An Arduino Micro controlling six Pololu DRV8835 motor drivers is used to supply current directly to the 12 TCAs that make up the legs, and a Pololu Dual Channel motor driver (MC33926) supplies current to the heating elements and three TCAs that make up the SMMs. The tether is made of sixteen 1 m long strands of insulated 34 gauge magnet wire, and the common ground is made of a 1m length strand of 28 gauge copper wire. Each wire in the tether has a JST pin soldered to one side and a JST socket soldered to the other.

The Arduino program uses the serial monitor as an interactive controller by taking a keyboard input and outputting a corresponding sub-routine. For instance, keys 1, 2, 3, 4, 5, 6 independently actuate Left TCAs 1, 2, 3 and Right TCAs 1, 2, 3 respectively. The operator can independently activate the heating elements for the SMMs and all 15 TCAs (12 for the legs and 3 for the body), which is useful for testing each actuator and measuring its resistance. We built sub-routines for standing the robot up, laying it down, mounting a beam, crawling, horizontal climbing, and walking using various gaits. Having each TCA on a pulse width modulation (PWM) pin enables us to tune the voltage it receives so that we can mitigate minor discrepancies in TCA resistance. Since an Arduino Micro only has 7 PWM pins, we used the "SoftPWM" (https://github.com/bhagman/SoftPWM) library, which allows a user to simulate PWM on any pin.

Supplementary Note 4.3. Foot trajectory and gait control

After assembling the robot, we began to realize morphing from one body type to another and executing various gaits and modes of locomotion, starting from the simplest function of crawling and moving on to progressively more difficult ones: walking and horizontal climbing. For

each gait, the foot trajectory is the same for the four legs, but the gait patterns are different, as shown in Supplementary Fig. 11A.

The simplest of the three modes of locomotion that we could execute is crawling. We define crawling as a static gait because dynamic forces such as momentum and inertia do not affect the motion and because each step cycle can occur independently. To enable crawling, the robot must be in a lying-down body configuration. When heated, the shape memory polymer's recovery force and gravitational force will morph the robot body into a lying down configuration from standing or gripping a beam. It is important that the legs are not bent so far that the feet cannot lift completely off the surface, as that would impede body movement.

To realize the crawling gait, all four legs move simultaneously according to the pattern as shown in Supplementary Fig. 11A. The numbering of the TCA and their position relative to the body is shown in Supplementary Fig. 11B. TCAs are being actuated during each time step of a cycle. One cycle is split into six 600 ms time steps (Supplementary Fig. 11C). For crawling, the first-time step has TCA1 and TCA3 actuated which acts to push the feet down towards the crawling surface and lift the robot body up. Next, only TCA3 is actuated for two-time steps to push the foot down and back, propelling the robot forward. Next, only TCA2 is actuated to lift the leg up and allow the robot body to rest back onto the crawling surface. During the following time step, TCA2 and TCA1 are actuated to lift the robot's foot off the surface to step forward. Finally, TCA1 alone is actuated bringing the foot to its furthest forward position, before the cycle starts over with a downward step. The trajectory is plotted in Supplementary Fig. 11D. The tether has very little effect on the crawling gait as long as it can move freely with the robot.

The walking mode of locomotion is more difficult to execute than crawling because it is a dynamic gait, where cycles have to be executed continuously and the robot has to maintain balance during the process. The robot must be in a standing configuration to walk. The robot has to start in a lying-down configuration before executing a stand-up maneuver (to go from

the horizontal climbing grip to standing it must lie down first). The ideal SMM body angle for walking is between 110° and 150°. If the body bending angle is higher than 150°, the robot base is so small that the robot will lose balance and fall over during a step. If the body angle is lower than 110°, the legs are being loaded transversely with too much reaction force from the walking surface and are bent to the point where the TCAs cannot provide enough displacement to lift the feet off of the surface during steps.

The walking gait is more complex than crawling because two pairs of feet have to work in synchronization, but with offset cycles. To maintain balance and symmetry throughout the dynamic movement, we break each foot's step cycle into evenly spaced time steps and offset the two pairs' step initiation by a half cycle. You can see the rear-right and front-left legs are a synchronized pair, as are the rear-left and front-right pair. Further, TCA actuation during the first half-cycle for one pair is identical to the second half-cycle of the other pair and vice-versa. This gait pattern is commonly known as a trot gait (Supplementary Fig. 11B). Fundamentally, the way this gait works is while one diagonal pair is lifting up and stepping forward, the other pair is pushing down to the walking surface to prevent the robot from sagging while the robot's entire weight is momentarily distributed across only two legs. Additionally, the cycle time has to be short enough that the robot does not tip while it is standing on two legs.

The horizontal climbing mode of locomotion is the most difficult to realize. It combines gripping a beam with a bound-type inching gait. The working principle of the horizontal climbing mode is the robot body will bend to an angle of approximately 180° so that the legs effectively grip the beam. The feet must be supported vertically, but the ledge of the beam (a piece of glass) does not contribute to the movement. The forward motion is produced when the robot releases the grip of two legs, reaches forward, then grips again and pulls itself forward. Note that, as shown in Supplementary Fig. 11C, we add extra actuation for TCA2 for the third and the sixth cycles. Using the SMMs to achieve a gripping force is difficult because the TCAs must

be continuously actuated while the body cools, but if they are even 5° C too hot, the actuation force produced onto a rigid body will be high enough to catastrophically damage the TCA.

The horizontal climbing gait is similar to the walking gait in that there are two pairs of synchronized legs operating a half-cycle out of sync with each other. The difference is that this gait uses the bound gait pattern (Supplementary Fig. 11A), where the front two legs are one pair and the back two legs are the other. Supplementary Fig. 11C illustrates the actuation pattern for the horizontal climbing gait. You can see that, like walking, each cycle is symmetrical at the midpoint. This is crucial for movement because it means that when one pair of legs is gripping and pulling the robot forward, the other is releasing its grip allowing the robot to slide freely.

The difference between the two gaits can be further explained by the primary force propelling the robot forward. In walking, the robot leverages the friction force from the ground to move forward. In contrast, during horizontal climbing, the robot's forward movement relies on the friction force generated by actively clamping on the beam. To understand the importance of this friction force from clamping, let's consider a scenario where there is no beam. During the horizontal climbing gait, the robot's front or rear two legs move at the same time, resulting in the robot cannot lift its legs alternately as it would during walking. In this case, all the legs will be in constant contact with the substrate, meaning there is no stepping up at any time. Given that the front legs and rear legs move in opposite directions, any friction force from the ground (glass) will essentially cancel out, making forward motion impossible. Therefore, we know that it is the friction force from the beam that facilitates the robot's movement during horizontal climbing. This friction force exists because the robot clamps onto the beam after the shape-morphing process (the distance between the robot's legs is 56 mm if not clamped onto the beam, which is slightly less than the beam's width of 65 mm).

Supplementary Note 5. Shape-morphing amphibious robot (SMART)

The Shape Morphing Amphibious RoboT (SMART) uses a basic robot body shape with the design emphasis put on the efficient use of the SMMs. SMART can swim in water with flattened modules as well as walk on land with curved modules.

Supplementary Note 5.1. Mechanical design

The SMMs for SMART are built similarly to the SMMs used in previous sections; however, they have a few key differences to enable morphing in harsher and colder conditions. The SMMs for SMART use a shape memory polymer composed of 59% Epon-828 and 41% Jeffamine D-400 by weight. This is done to make the modules more ductile while cooled and lower their glass transition temperature to 55 °C. As SMART is untethered, using nichrome wire for heating would be impractical with the limitations of the battery due to its large resistance. With this consideration, the same conductive nylon thread used to create the TCAs is used to act as the resistance wire for these modules. The last change made to these modules is the use of dielectric clear grease (part NO. 05113, CRC Industries, Inc.) to coat the outside of the spines. This grease is used to allow the modules to better insulate themselves from harsh winds and cold waters to allow for morphing in environments that adversely affect the SMMs' actuation.

The SMMs are connected to SMART through a small pressure-fit clip designed into an SLA-printed shaft connector (Fig. 6b). This connector pairs with a gasket made of Ecoflex-50 and is slotted into the shaft connector. Each shaft connector is pressure fit to a motor shaft deeper within the robot body. As the shaft connectors are attached, the shaft connector and the gasket are thoroughly coated in dielectric silicon grease to ensure the robot is waterproofed.

Supplementary Note 5.2. Electrical system

SMART utilizes Bluetooth control and an 11.1-volt 3-cell lipo battery to allow for untethered actuation and control. It has a 5 V logic circuit accomplished with a 5 V step-down regulator

and an 11.1-volt circuit for the actuation of motors, TCAs, and heating (Supplementary Fig. 12). An Arduino MKR WiFi 1010 is used as the main controller. The morphing modules of SMART are controlled using PWM for the heating and TCA actuation. Each leg is driven by a 380:1 Pololu 6 V DC motor, leading to four motors in total, each with an encoder for precise rotational control.

Supplementary Note 5.3. Motion control for amphibious locomotion

Locomotion is enabled by multi-encoder positioning. For swimming, only the rear legs are used (the front legs are used for stabilization so that the robot can swim relatively straight). The swing angle for the rear legs with respect to time is shown in Supplementary Fig. 13. For morphing, the modules are first lifted out of the water in order to make heating easier. For walking, a “bound” gait is used, which is similar to the last figure in Supplementary Fig. 11A. As in Supplementary Fig. 13B, the front two legs are first rotated under SMART to act as rails to reduce friction. The rear modules then rotate 180° to propel SMART forward and place them underneath SMART to act as a secondary set of rails. The front legs are then rotated 180° and the cycle repeats.

A push-off movement enables SMART to swim off of rocky surfaces. The rear legs are rotated downwards at a 70° angle such that they slightly lift SMART off of the surface it’s on. This first movement is intended to lower the amount of the robot’s surface area that is in contact with ground surfaces. The front legs are then made to complete a full rotation forward in order to slide the robot off the surface. The modules do not need to be fully flattened in order for the robot to swim, but the bending angle must be less than 20 degrees; otherwise, SMART will be unable to achieve the thrust necessary for swimming.

Supplementary Note 5.4. Energy Consumption Estimation

The energy consumption for a single shape-morphing process is around $E_{total} = 350$ J for the 2D bending shape-morphing module when bending 90° s, which is used by our amphibious morphing robot. This energy is calculated using the same method in Supplementary Note 3.3.

Energy of the Battery: Next, we calculate how far our amphibious can move or how many morphing it can finish with the equipped battery. The battery (TA-45C-450-3S1P-JST) used in our amphibious robot is a 450 mAh 11.1 V battery. We can calculate the joule capacity of the battery by converting the mAh capacity into Joules resulting in a capacity of 17982 J.

Since it takes ∼350 J to morph one leg module, it takes ∼1400 J to morph all four legs. This indicates that the current battery can morph the legs six times *before being at half capacity*.

Energy for locomotion: Due to the negligible power draw from the onboard electrical components and zero power draw of the shape morphing scheme, the energy consumption during locomotion can be estimated by the power draw of the motors. We use 380:1 Micro Metal Gearmotors (Pololu item #: 4796) in the amphibious robot. It has an average current draw of 0.40 A with a nominal voltage of 6 V. Based on this, we can roughly calculate the run time of the amphibious robot, and therefore the traveling distance in water and on land respectively based on the running speed of the robot.

Specifically, we can calculate the time that a single motor can be run off of a 450 mAh, 11.1 V battery to be roughly two hours: Time=E/(V*A)=17982 J/(6 V * 0.4 A) =7492.5 s

We divide this by two as the total draw is doubled using two motors (only two motors are active at any given time) for terrestrial locomotion and get a run time of roughly 62 minutes for terrestrial locomotion. If we assume that below half charge, the battery will not sufficiently power this locomotion, resulting in a run time of 31 minutes.

For aquatic locomotion, there is a delay between the movements of the legs, but at any given time, two motors are used in unison. There is a delay of 0.1 seconds between up and down movements for the legs to allow the robot to coast through the water (0.2 seconds of any

given cycle is spent on this pause). This means that aquatic locomotion can be actuated for 1.2 times the amount of time as terrestrial locomotion, i.e., 31 mins * 1.2 = 37.2 mins.

These values can be used to estimate the actual distance the amphibious robot can move for aquatic and terrestrial locomotion by multiplying the speed and the run time, respectively. The terrestrial distance (D_t) can be found by: D_t=31 min * 60 sec/min * 1 BL/sec * .14 m/body =260.4 m. The aquatic distance (D_a) can be found similarly: D_a=37 min * 60 sec/min * 0.2 BL/sec * .14 m/body=62.2 m. Therefore, we estimate the operational time to cause a fully charged battery to reach half capacity as follows: the robot can do the full morphing 6 times for four legs, or walk for 31 mins (distance 260.4 m), or swim for 37 min (distance 62.2 m).

Supplementary Note 6. Elementary Shape-morphing Modules

The twisting module consists of an SMP spine, several rings, and a sheathed TCA (Supplementary Fig. 14A). The spine has a center rod (diameter 1.2 mm) and protrusions around the rod to connect the rigid rings. A resistance wire (36 AWG) is wrapped around the center rod to apply Joule heating. Each ring is connected with three protrusions around the rod. The rings are fitted together by flanges that only allow the rotational motions around the center rod. The sheathed TCA is wrapped around the rings, and the two ends are fixed to the top and bottom rings respectively. The purpose of the rings is to 1) form a cylindrical surface to hold the sheathed TCAs; 2) get rid of the bending, shear, and axial compression to only leave the torsional freedom.

The 3D bending module has a spine similar to the twisting module that consists of a center rod and protrusions (Supplementary Fig. 14B). Three sheathed TCAs are glued to the three groups of protrusions on the center rod. To glue the sheathed TCA onto one group of protrusions, 3D-printed jigs are used to hold the sheathed TCA and the spine. A resistance wire (36 AWG) is wrapped around the center rod to apply Joule heating. Each sheathed TCA is individually controlled to morph the module in three-dimensional space.

Similarly, the twisting and bending module is fabricated by arranging the TCAs in a ZigZag shape and glued to the protrusion on the spine (125×10×5 mm). The process is facilitated by a 3D-printed jig to fix the sheathed TCA in the Zigzag shape. A prestretch is inserted into the elastic sheath of the TCA to balance the gravitational force of the module, which will increase the module's deformation. That is to use 50% of the required length, which is the length of the groove in the jig. After the TCA is placed in the sheath, the sheathed TCA is stretched to be placed in the groove. The inclined angle of the sheathed TCA will directly influence the bias angle of the helical shape, and the extreme case is when the inclined is zero, which becomes the 2D SMM that does not have any twisting and bends to a circle.

The discrete surface module consists of a rectangular surface (80×60×1.15 mm without considering the protrusion), resistance wires, 3D-printed tubes, and a sheathed TCA (Supplementary Fig. 15A). 3D-printed PETG tubes are pressure-fit onto the protrusions around the surface base. A sheathed TCA is threaded through each tube with the start and end of the TCA meeting at the bottom of the surface base. This TCA is then secured by the start and end points to the tubes using silicon epoxy (Silpoxy). This TCA can be controlled to actuate the module once creases are heated. When the discrete surface bends, the soft creases will have very large curvature (strain) due to the small width of the creases. Therefore, we use a different SMP that can have a larger maximum strain. It is made from a 38% Jeffamine d400 to 62% Epon 828 mixture by weight. This mixture is formed into an SMP base through the use of a silicon mold made with DragonSkin-30 (Smooth-on, Inc.). A top piece is placed on the mold to ensure a uniform surface. Once in the mold, the SMP mixture is left to cure for 12 hours at 72 °C. The resulting SMP surface base is durable but has the ductility needed to support small bending angles. Flat, 0.70 mm wide Kanthal A-1 resistance wire is then embedded into grooves molded into the surface base. This wire is then secured using silicon glue (Sil-Poxy, Smooth-on Inc.) by filling the remaining area of the crease. This is repeated five times to form each crease.

This forms a matrix of possible folding areas on the discrete surface module. The ends of the resistance wires for each joint are then soldered into wires connecting them to high-amperage MOSFET motor drivers.

The grid surface consists of a network of 5 horizontal and 5 vertical beams ($80\times80\times8$ mm) as shown in Supplementary Fig. 15B. It is fabricated with the same molding method as in Supplementary Note 1. 2.. Each beam is wrapped with a resistance wire (not shown in the schematic). There are protrusions at the intersections of beams (a total of 25). Connectors are used to easily arrange the TCA in a square loop around the four edges of the grid surface. The connectors are 3D-printed with an SLA printer and can be pressed to fit into the protrusions. The four TCAs are individually controlled to morph the shape of the grid surface.

Supplementary Figures

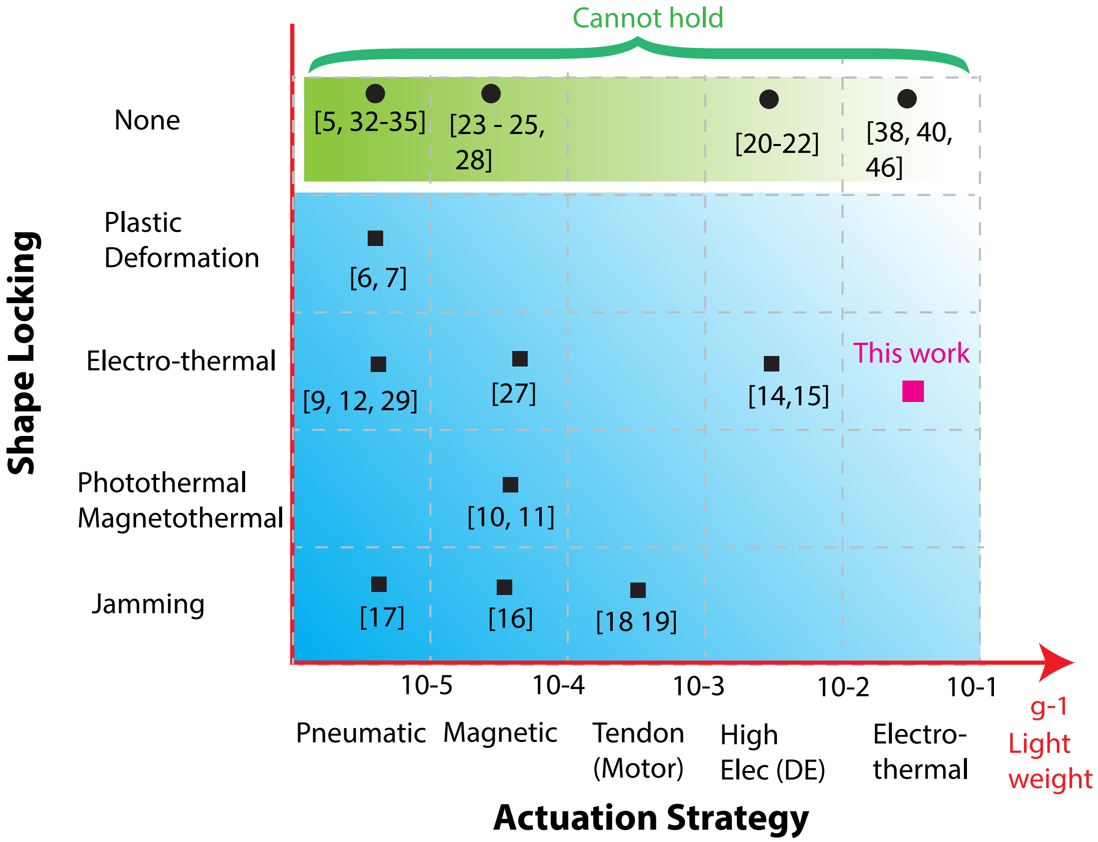

Supplementary Fig. 1. Comparison of our work with existing shape-morphing schemes. The horizontal axis represents 1/weight: i.e., if the weight for the scheme's actuation strategy is smaller, then it will approach to the right. Note that it is difficult to obtain exact numbers for different strategies, and we roughly categorize them into the figure. The vertical axis categorizes the shape locking methods. Note that the schemes at the top of the figure cannot hold the shape without continuously applying actuation.

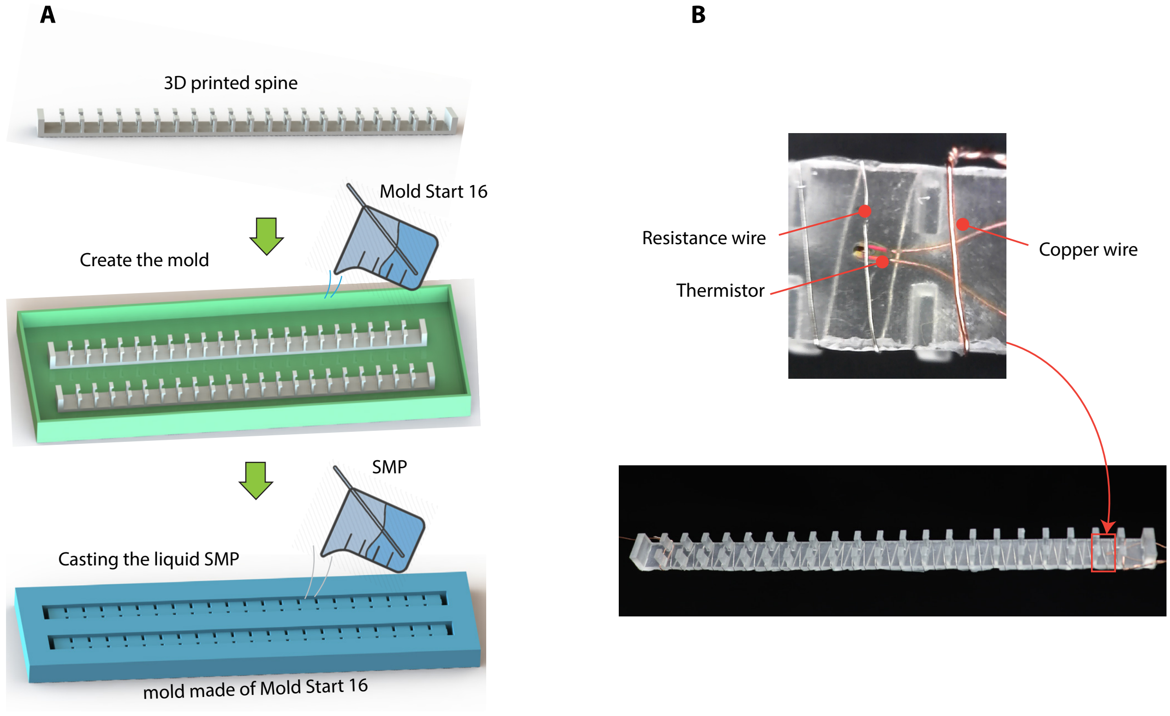

Supplementary Fig. 2. Fabrication of the SMP spine for the 2D bending SMM. (**A**) The shape of the spine is 3D printed with a Prusa SLA SL1S printer. The mold is created by pouring Mold Start 16 (Smooth-on Inc.) into a container with the spines placed on the bottom. The mixed SMP liquid is poured into the mold and degassed in a vacuum oven. A flat clear piece made of SORTA-Clear (Smooth-on Inc.) is placed on the top to enclose the SMP and the SMP is cured at 75 °C for 12 hours. (**B**) The SMP is wrapped with a resistance wire (Nichrome, AWG 38). The zoomed-in picture shows the embedded thermistor.

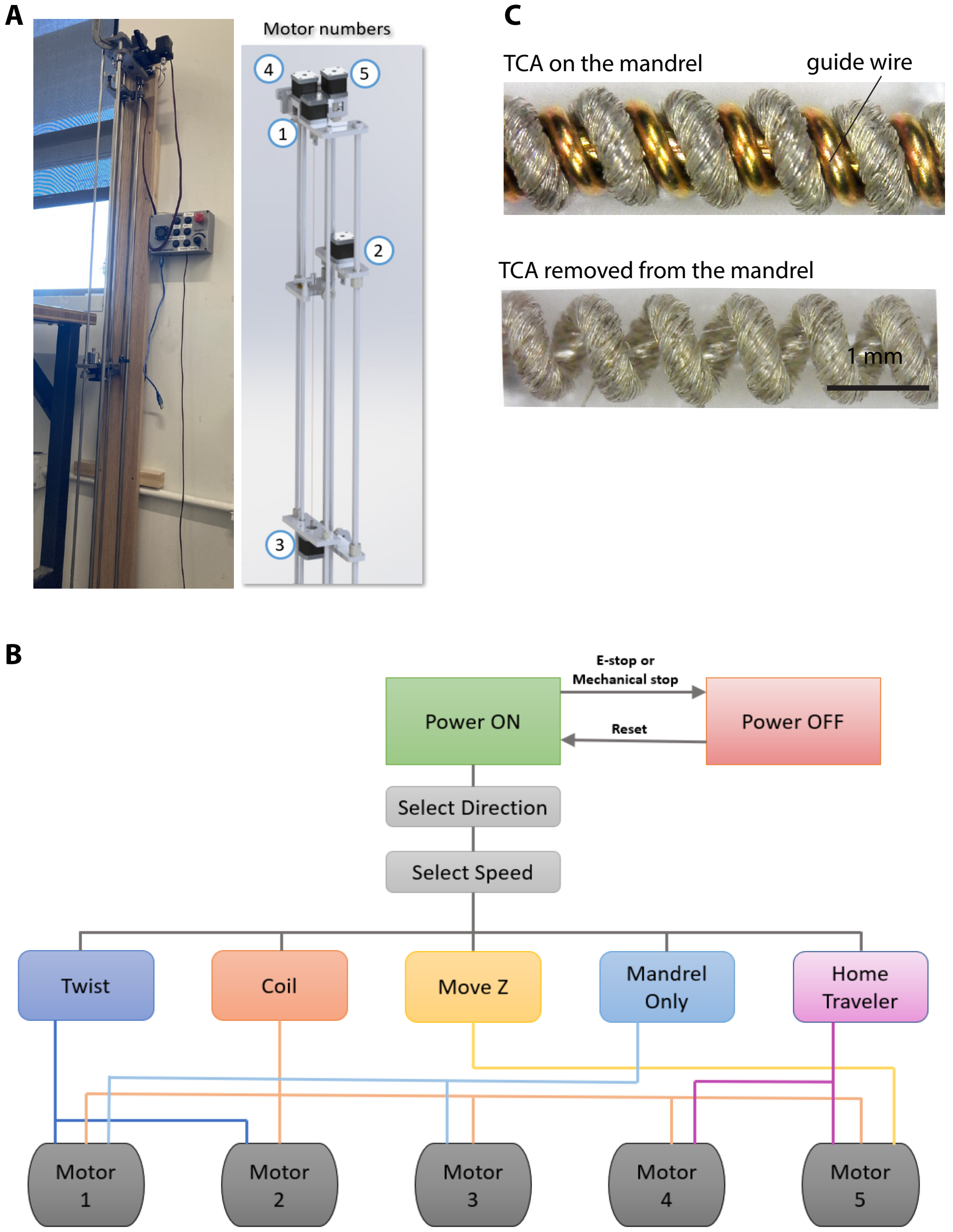

Supplementary Fig. 3. Fabrication of the TCA. (**A**) A photo and schematic of the customized machine. The numbers are for labeling the motors. (**B**) The control schematic of the machine. There are a total of five motors to first twist and then coil the twisted thread. (**C**) The TCA after annealing on a helical mandrel and after removing the mandrel.

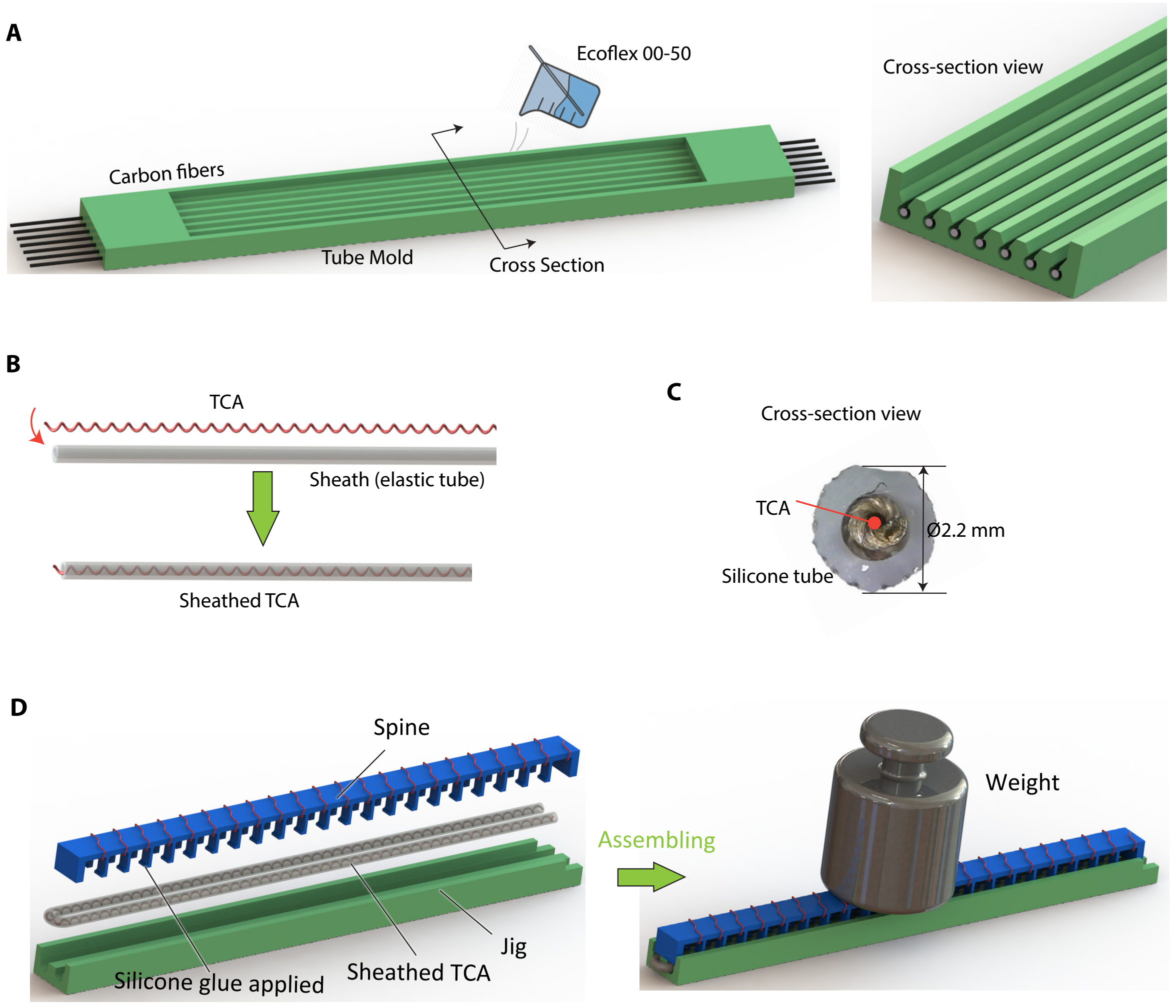

Supplementary Fig. 4. Fabrication of the sheathed TCA and assembly of the sheathed TCA and the spine (**A**) The 3D printed mold fabricates the elastic tubes as the sheath. It can fabricate seven tubes at a time. The figure on the right shows the cross section of the mold. (**B**) The sheathed TCA is fabricated by inserting the TCA into the elastic tube. (**C**) The cross section is shown and the outer diameter of the sheathed TCA is 2.2 mm. (**D**) To assemble the sheathed TCA and the spine, a jig is used to hold the TCA in a U shape and fix the spine in place (left). A weight is placed on the top of the spine when the glue is curing (right).

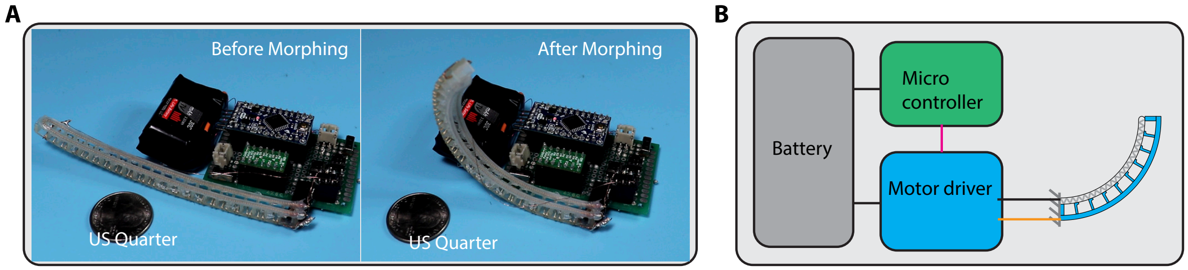

Supplementary Fig. 5. The minimal system. (**A**) The minimal system before and after morphing. (**B**) The block schematic of the minimal system.

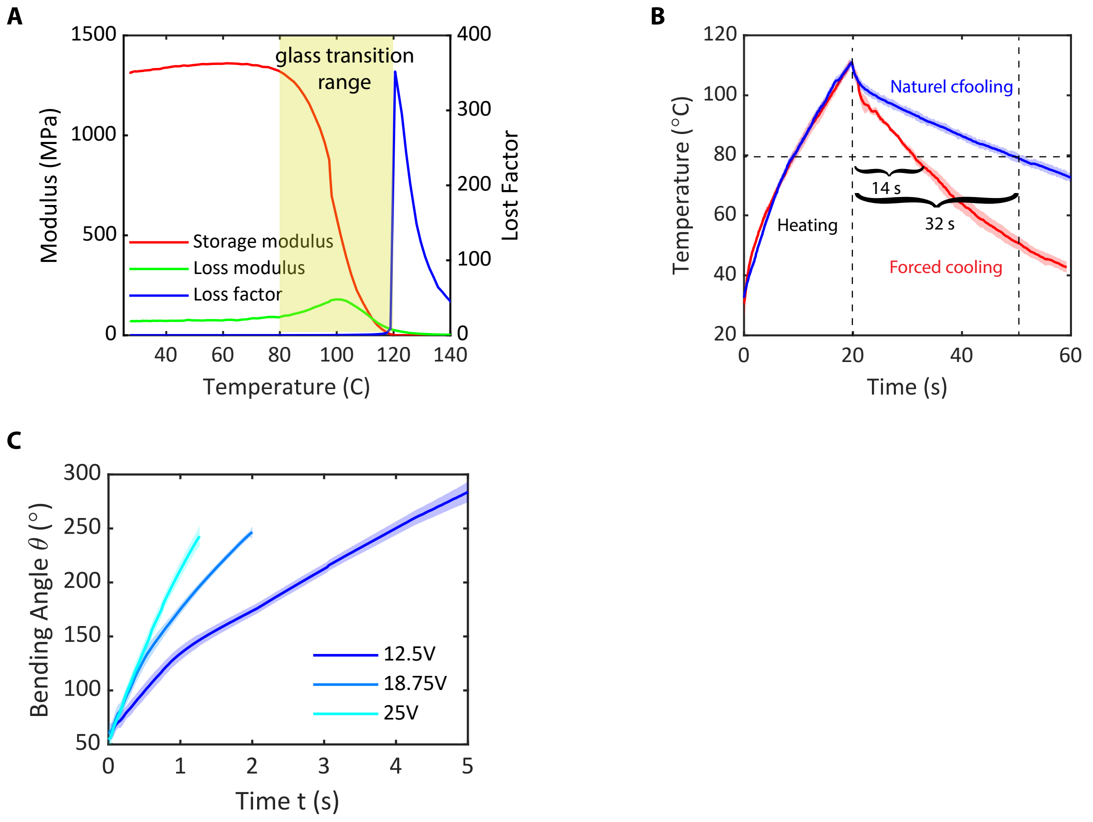

Supplementary Fig. 6. Characterization of the 2D bending SMM. (**A**) The SMP's storage moduli (E'), loss moduli (E''), and loss factor (tan δ) through dynamic mechanical analysis (DMA) test. The glass transition temperature T_g is around 100 °C. (**B**) The temperature of the spine with natural cooling and forced cooling. (**C**) The bending angle of the 2D bending module with respect to time for quick actuation.

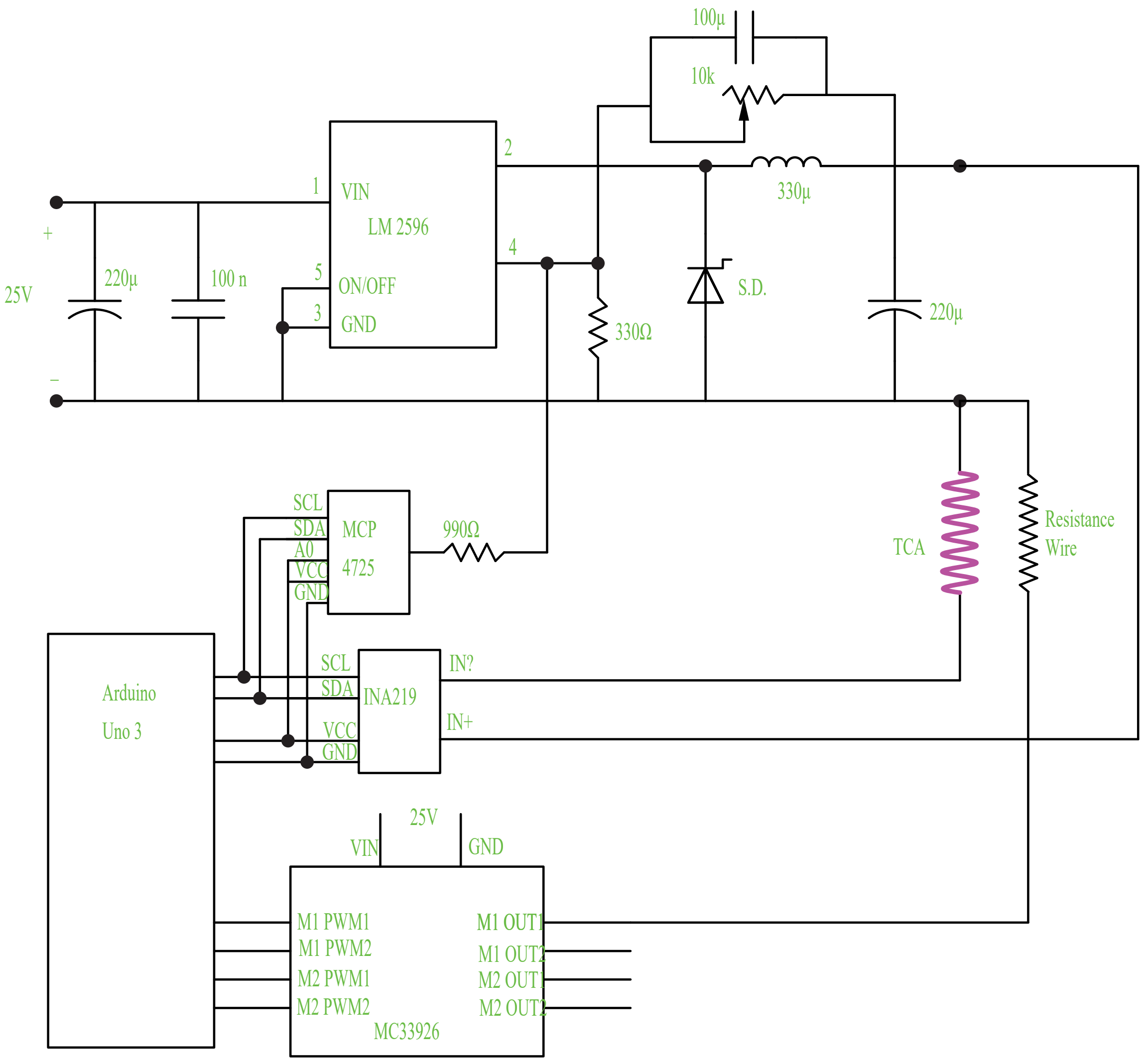

Supplementary Fig. 7. The circuit for the closed-loop control. The circuit is used to measure the resistance of TCAs with respect to a 2D bending module's bending angle and it is also used to conduct the closed-loop control of modules.

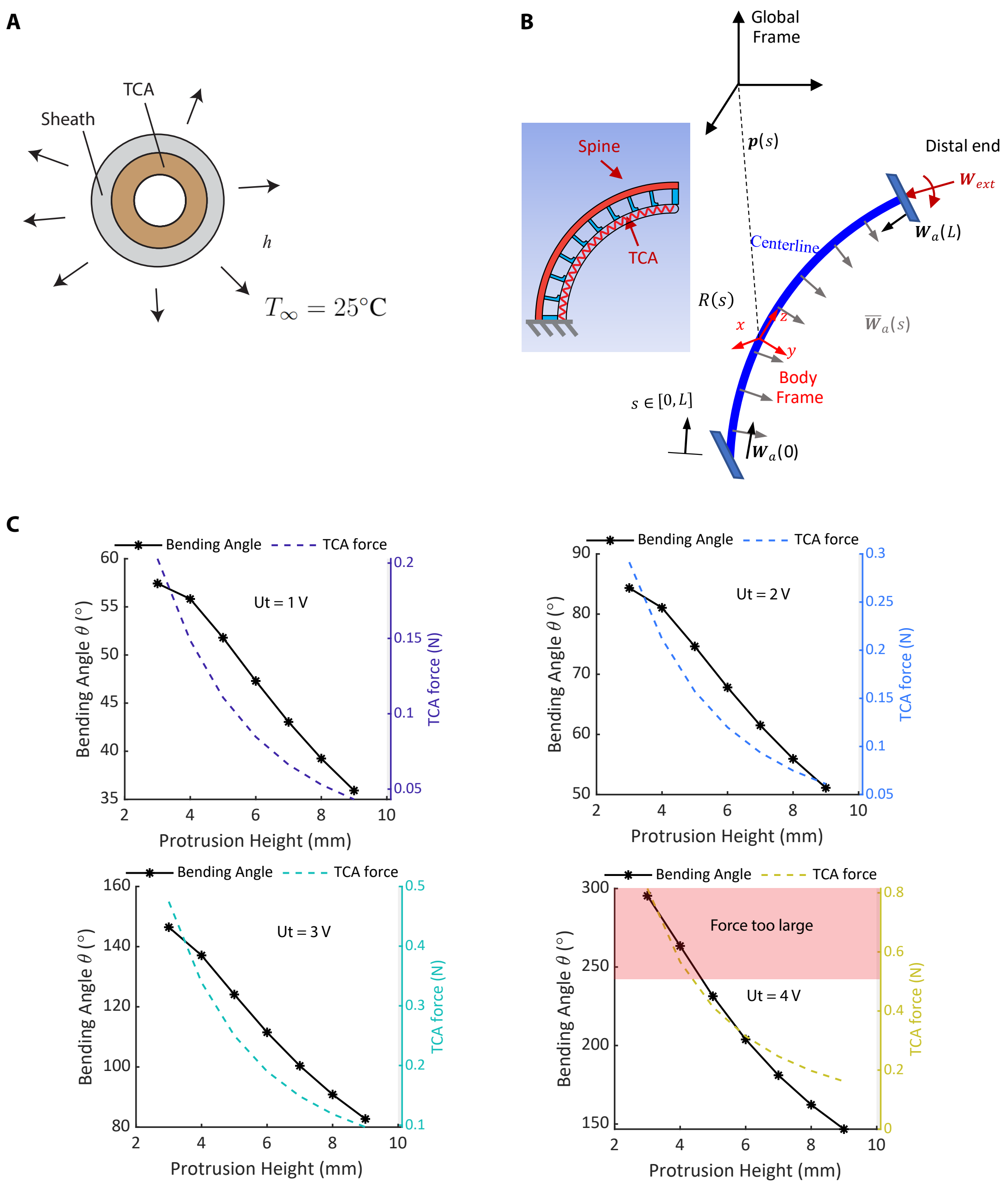

Supplementary Fig. 8. The schematics and results for the analytical model. (**A**) The schematic for the thermal model of a sheathed TCA. (**B**) The spine is modeled using the Cosserat rod model. (**C**) The bending angle for the SMM and the TCA force when varying the height of the protrusions from 3 mm to 9 mm for different but constant input voltages.

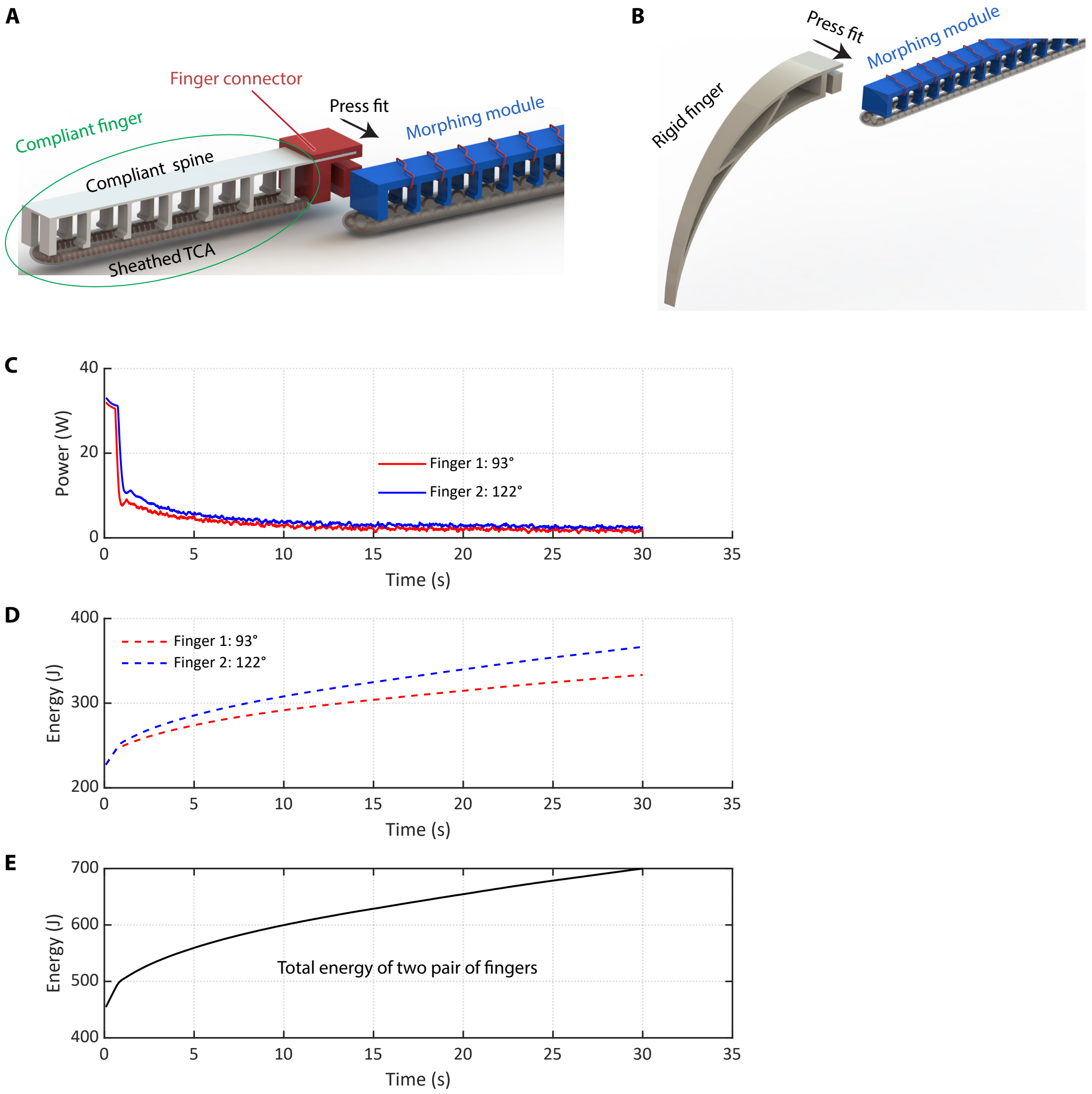

Supplementary Fig. 9. The design of the shape-morphing grippers. (A) For shape-morphing gripper 1, the 3D-printed spine of the compliant finger is connected to the shape-morphing base using a connector. (B) For shape-morphing gripper 2, the rigid gripper is directly 3D printed and connected to the shape-morphing base. (C)-(E) The power and energy consumption plots of the shape morphing gripper 2 for caging a mouse with respect to time. (C) The power of the two pair of fingers. (D) The energy consumption of the two pairs of fingers. (E) The total energy consumption of the two pairs of fingers.

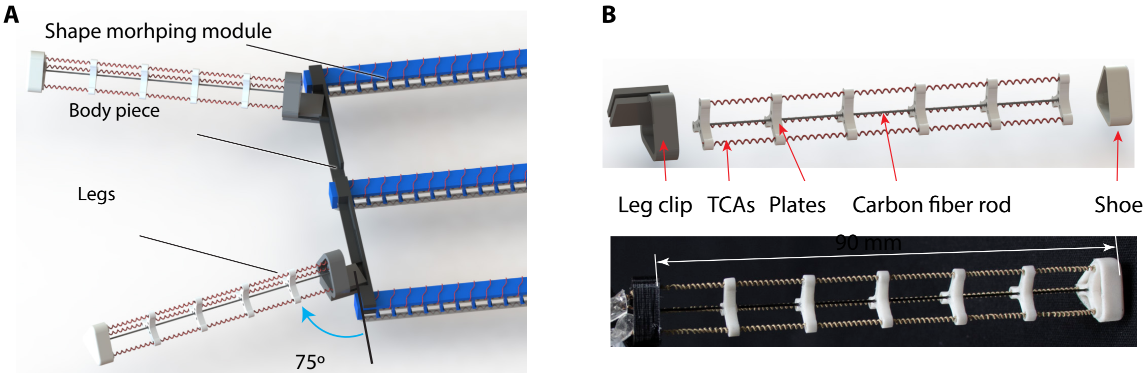

Supplementary Fig. 10. Design of the shape-morphing quadrupedal robot. (**A**) The assembly of the robot includes three SMMs, two body connectors, and four legs. The leg has an angle of 75° with respect to the body piece. (**B**) The assembly of the continuum leg has three TCAs running through the holes in the plates.

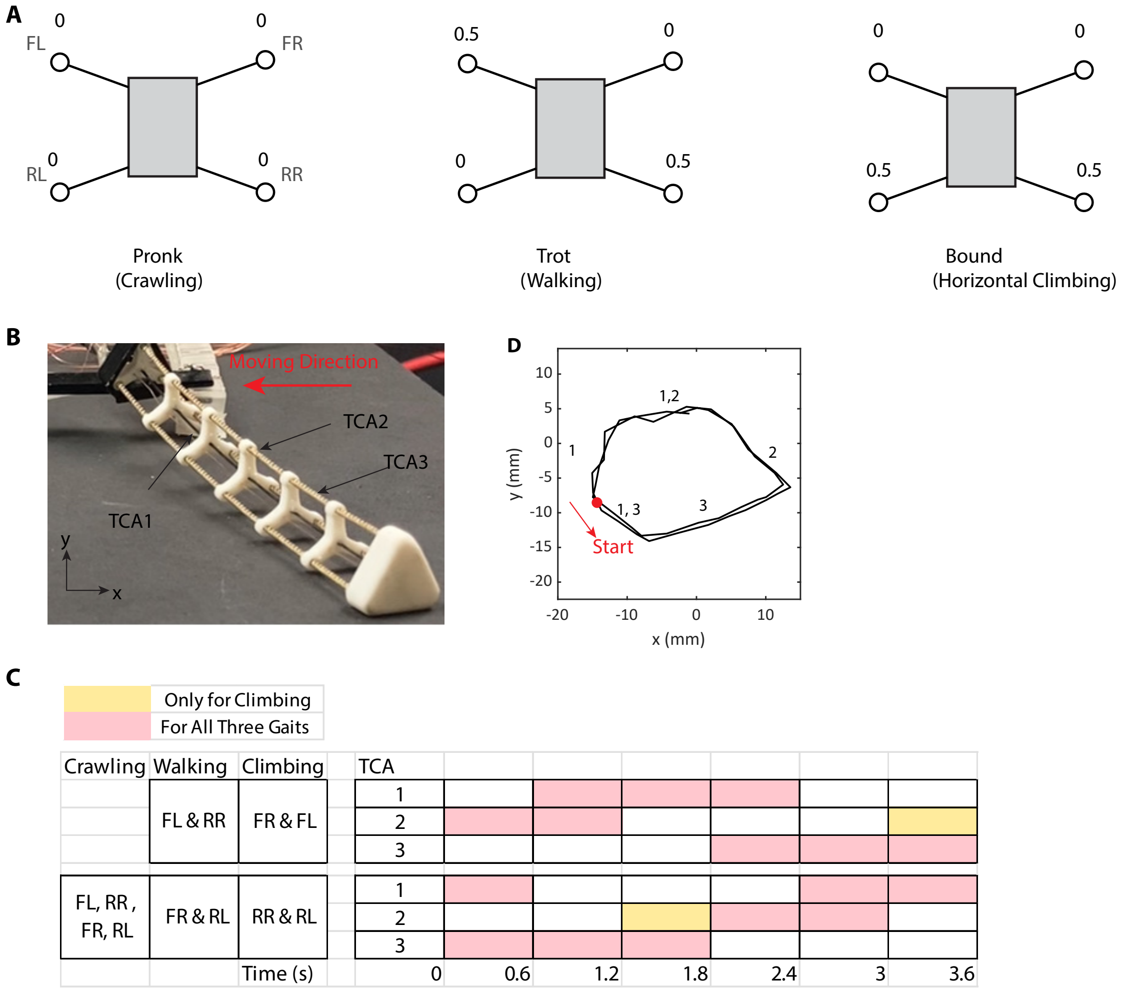

Supplementary Fig. 11. Locomotion design of the quadrupedal robot. (**A**) The gait patterns of the three locomotion types. The values shown around the feet represent the typical phases relative to the front left (FL) leg. (**B**) The numbering of TCA. (**C**) The actuation sequences of TCAs for crawling, walking, and horizontal climbing. (**D**) The foot trajectory of the robot for crawling.

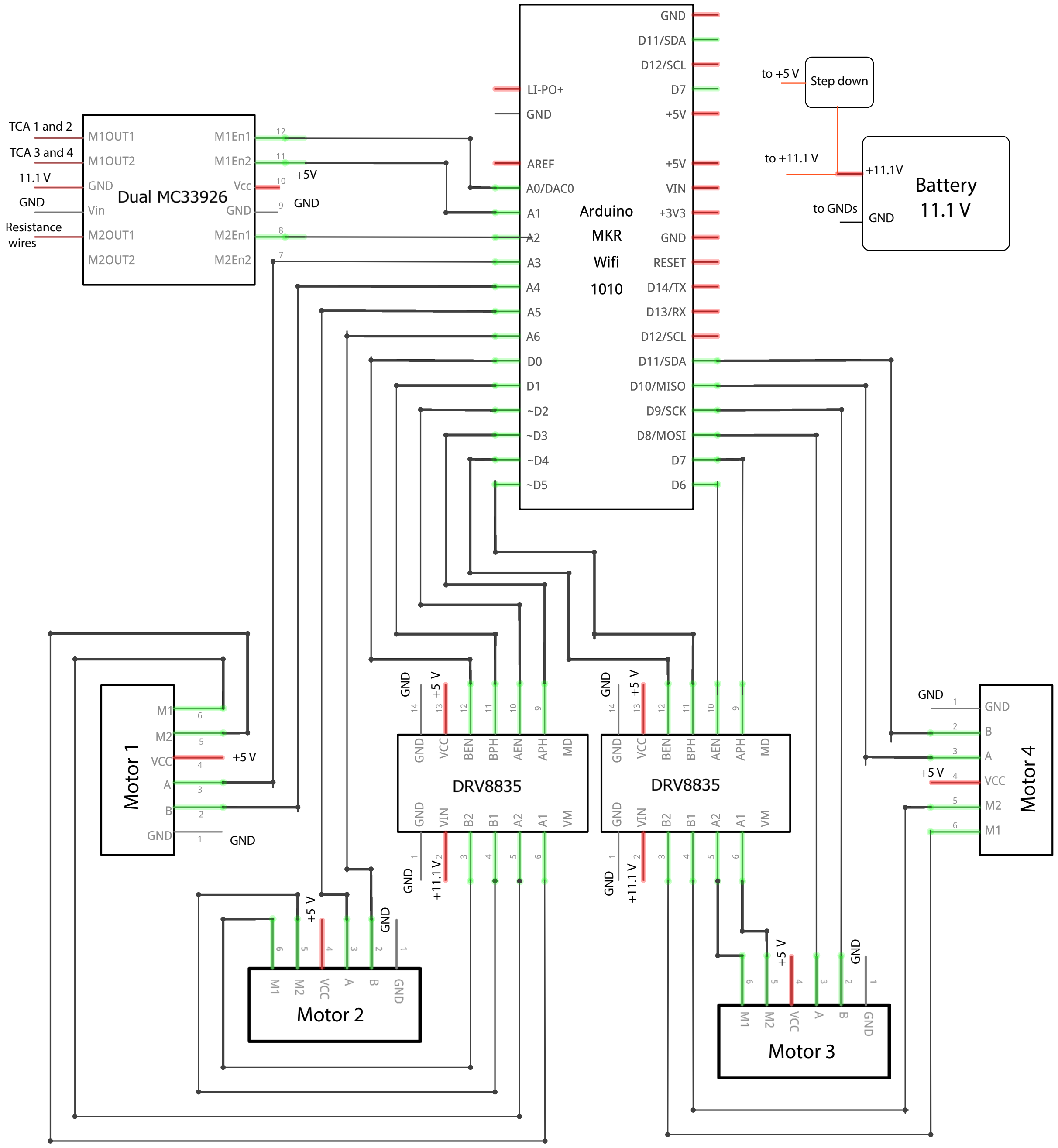

Supplementary Fig. 12. The schematic of the electric circuit for the amphibious robot.

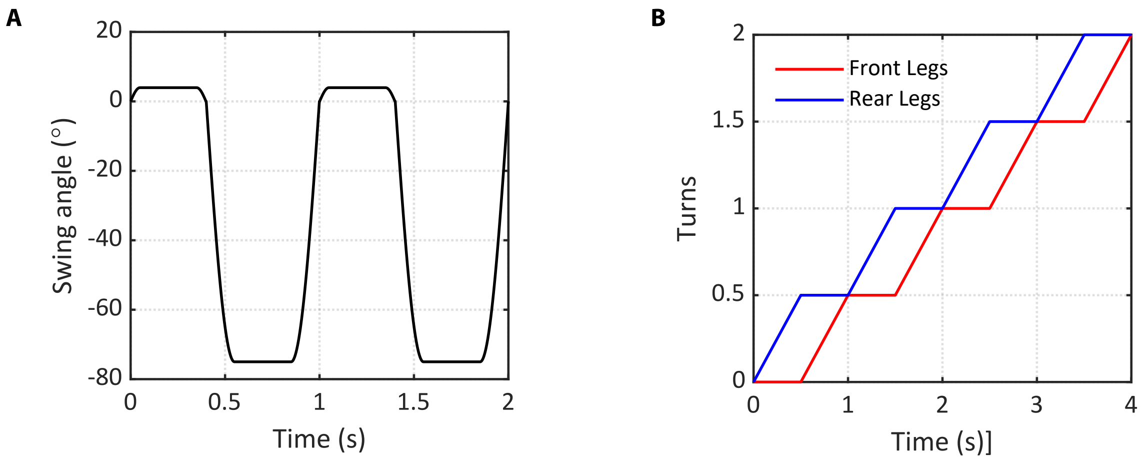

Supplementary Fig. 13. The gait of the amphibious robot. (**A**) The swing angle of the rear legs with respect to time for swimming. The figure shows two cycles. (**B**) The turns of the front legs and rear legs for walking. The figure shows two cycles.

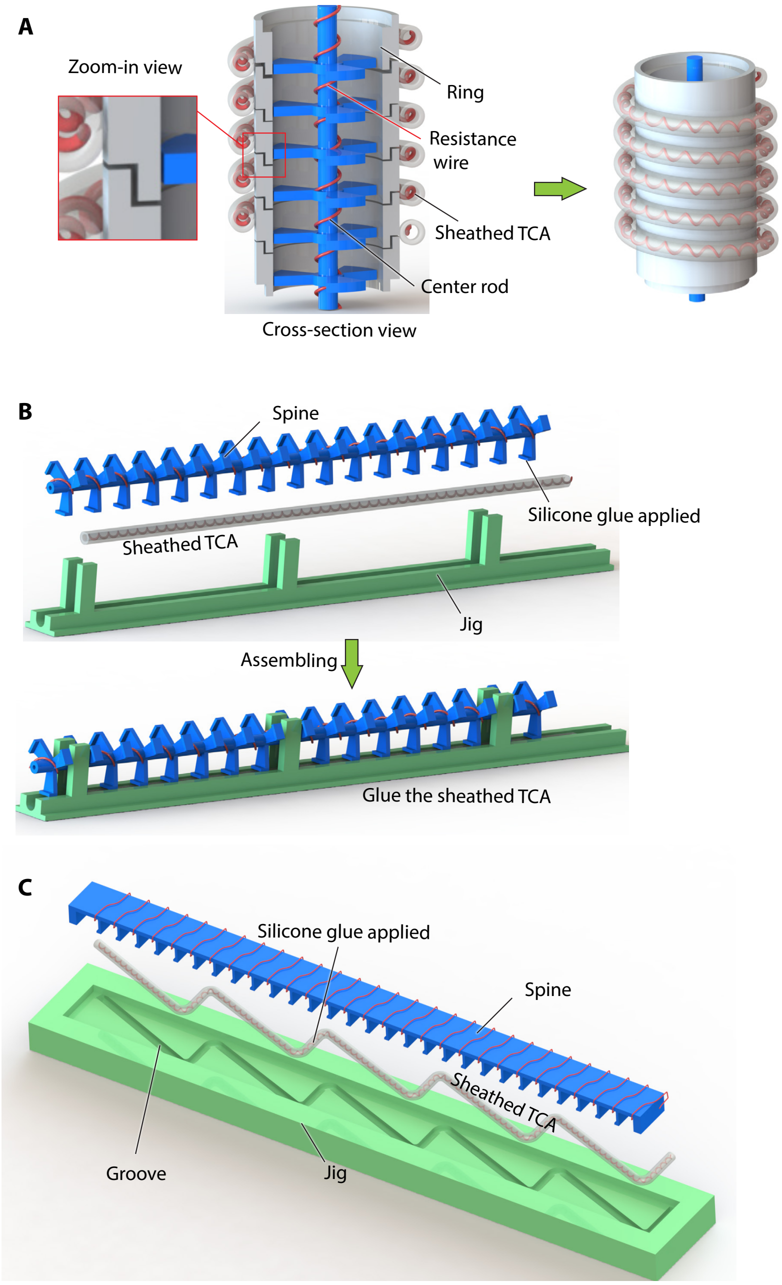

Supplementary Fig. 14. The design of the twisting, 3D-bending, and twisting and bending module. (**A**) The twisting module has a few rings assembled together and neighboring rings can fit into each other to leave only the rotational degree of freedom as shown in the zoom-in view. (**B**) A jig is used to fix one TCA and the spine in a straight shape to assemble the TCA. (**C**) A jig is used to fix the TCA in a Zigzag shape and assemble the TCA to the protrusion on the spine.

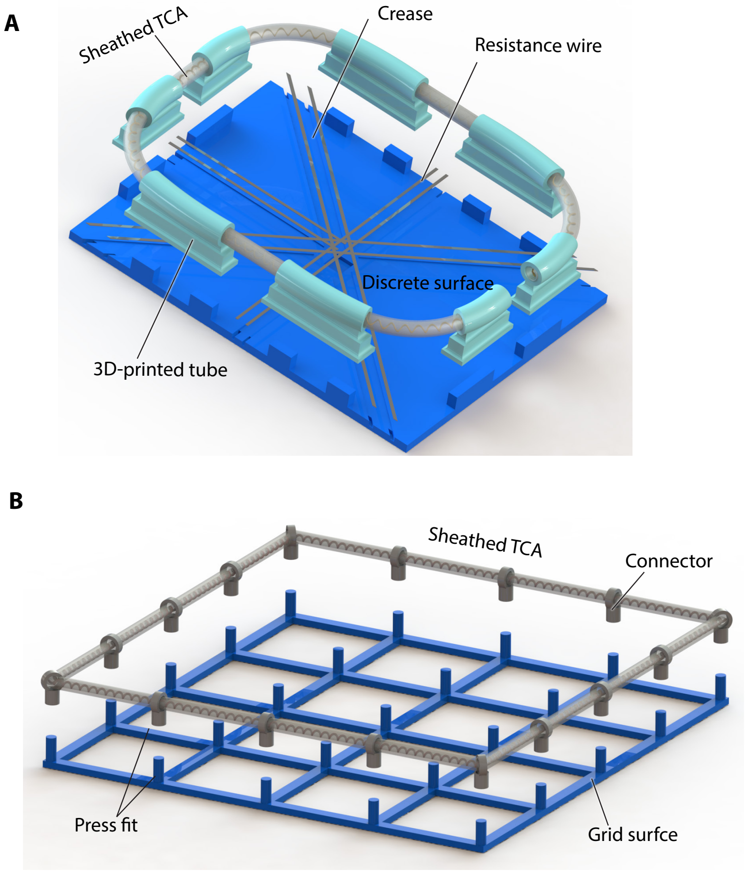

Supplementary Fig. 15. The design of the discrete and grid surface module. (A) The design of the discrete surface module. (B) The design of the grid surface module.

Supplementary Table

Supplementary Table 1. Comparison of shape-morphing schemes in existing studies

Ref.	Active materials	Actuation	Embedded actuation	Embedded sensing	Embedded locking	Local control
**This Work**	**Nylon (TCA)**	**Electricity**	✓	✓	✓(SMP)	✓
(14)	DEA	Electricity	✓	×	✓(LMPA)	×
(15)	DEA	Electricity	✓	×	✓(SMP)	✓
(20,21)	DEA	Electricity	✓	×	×	×
(22)	DEA + LCE	Electricity	✓	×	×	×
(16)	Ferromagnetic	Magnetic	×	×	× (Jamming)	×
(10)	MRE	Magnetic	×	×	× (Photothermal)	×
(11)	MRE	Magnetic	×	×	× (SMP, mag-heating)	×
(23–25)	MRE	Magnetic	×	×	×	×
(26)	MRE + LCE	Magnetic + heat	×	×	×	✓
(27)	MRE	Magnetic	×	×	✓(SMP)	✓
(28)	Nanomagnets	Magnetic	×	×	×	×
(17)	Elastomer	Pneumatic	×	×	× (Particle Jamming)	×
(12)	Elastomer	Pneumatic	×	✓	✓(Thermal plastic)	×
(29)	Elastomer	Pneumatic	×	×	✓(Thermal polymer)	×
(5, 32, 33, 35)	Elastomer	Pneumatic	×	×	×	×
(34)	Elastomer with tensile jamming	Pneumatic	×	×	×	✓
(7)	Elastomer	Pneumatic	×	×	✓(clay)	✓
(9)	Elastomer	Pneumatic	×	×	✓(SMP)	✓
(36)	Printable Ink (PDMS)	Heat (water)	✓	×	×	×
(37)	SMP	Heat	✓	×	× (SMP, coupled)	×
(38)	SMA	Heat (patten)	✓	×	×	✓
(44)	Hydrogels	Hydraulic	✓	×	×	×
(45)	Hydrogels	Heat (env)	✓	×	×	×
(46)	Hydrogels	Heat (Magnetic)	✓	×	×	×
(39)	LCNs (kirigami)	light	✓	×	×	✓
(40)	LCEs	Heat	✓	×	×	✓
(41)	LCE with LM	Heat	✓	×	×	✓
(42, 43)	LCE	Heat (water)	✓	×	×	×
(18, 19)	Tendon	Linear actuator/motor	×	×	× (Layer jamming)	×
(6)	\	External force	×	×	✓(plastic deformation)	✓
DEA: Dielectric elastomer actuator; LMPA: low-melting-point alloy; LCE: liquid crystal elastomers; LM: liquid metal; PDMS: Polydimethylsiloxane; MRE: magnetic responsive elastomer; LCNs: LCEs and polymer networks						
