# Supplementary material for: Embedded shape morphing for morphologically adaptive robots
Source: Nat Commun. 2023 Sep 27;14:6023. doi: 10.1038/s41467-023-41708-6 (PMC10533550; doi:10.1038/s41467-023-41708-6)
Supplement: Supplementary file 3 — Description of Additional Supplementary Files Document [file 41467_2023_41708_MOESM3_ESM.pdf]

Description of Additional Supplementary Files

Supplementary Movie Legends

Supplementary Movie 1. Shape morphing of a 2D bending module.

Supplementary Movie 2. Closed-loop control of a 2D bending module.

Supplementary Movie 3. Shape-morphing gripper 1.

Supplementary Movie 4. Shape-morphing gripper 2.

Supplementary Movie 5. Shape-morphing quadrupedal robot.

Supplementary Movie 6. Shape-morphing amphibious robot.

Supplementary Movie 7. Twisting module, 3D bending module, twisting and bending module.

Supplementary Movie 8. Discrete surface module and grid surface module.
